# The NSUN5-FTH1/FTL pathway mediates ferroptosis in bone marrow-derived mesenchymal stem cells

**DOI:** 10.1038/s41420-022-00902-z

**Published:** 2022-03-05

**Authors:** Jie Liu, Zhenxing Ren, Lin Yang, Lulu Zhu, Yi li, Caiqun Bie, Helu Liu, Yichun Ji, Dongfeng Chen, Meiling Zhu, Weihong Kuang

**Affiliations:** 1 Traditional Chinese Medicine Innovation Research Center, Shenzhen Hospital of Integrated Traditional Chinese and Western Medicine, Shenzhen, China; 2 Shanghai Jiao Tong University Affiliated Sixth People’s Hospital, Shanghai, China; 3 The First Dongguan Affiliated Hospital, Guangdong Medical University, Dongguan, 523710 China; 4 Shenzhen Bao’an Traditional Chinese Medicine Hospital, Guangzhou University of Chinese Medicine, Shenzhen, China; 5 School of Basic Medical Science, Guangzhou University of Chinese Medicine, Guangzhou, 510006 China; 6 Guangdong Key Laboratory for Research and Development of Natural Drugs, Key Laboratory of Research and Development of New Medical Materials of Guangdong Medical University, School of Pharmacy, Guangdong Medical University, Dongguan, 523808 China

**Keywords:** Cell death, Mesenchymal stem cells

## Abstract

Ferroptosis is a type of cell death induced by the iron-dependent accumulation of lipid hydroperoxides and reactive oxygen species (ROS) in cells. Inhibiting ferroptosis is important for improving the survival of transplanted bone marrow-derived mesenchymal stem cells (BMSCs). Although it is known that NOP2/Sun RNA methyltransferase 5 (NSUN5) post-transcriptionally regulates ferroptosis in BMSCs through RNA methylation, the precise mechanisms underlying these effects have not been reported. In this study, we demonstrate that NSUN5 is downregulated in erastin-induced ferroptosis in BMSCs. Ferroptosis was inhibited by the overexpression of *NSUN5* or ferritin heavy chain/light-chain (FTH1/FTL) and was enhanced by *NSUN5* knockdown. RNA immunoprecipitation experiments revealed that NSUN5 binds to FTH1/FTL, while *NSUN5* depletion reduced the levels of 5-methylcytosine in *FTH1*/*FTL* RNA and increased intracellular iron concentrations, resulting in the downregulation of glutathione peroxidase 4 (GPX4) and the accumulation of ROS and lipid peroxidation products. Co-immunoprecipitation experiments demonstrated that the recognition of *FTH1* and *FTL* by NSUN5 is dependent on the recruitment of tumor necrosis factor receptor-associated protein 1 (TRAP1). These results suggested that the NSUN5-FTH1/FTL pathway mediates ferroptosis in BMSCs and that the therapeutic targeting of components of this pathway may promote resistance to ferroptosis and improve the survival of transplanted BMSCs.

## Introduction

Mesenchymal stem cell (MSC) transplantation is an important treatment option for acute liver failure as well as for cardiac and neurodegenerative diseases [1–3]. Bone marrow-derived mesenchymal stem cells (BMSCs) can home to injured tissues and contribute to tissue repair [4]. Perturbation of the intracellular environment and metabolism are major causes of treatment failure in patients following transplantation. The latter can lead to iron overload and the toxic accumulation of reactive oxygen species (ROS) and lipid hydroperoxides, resulting in a form of controlled cell death known as ferroptosis [5]. Inhibiting ferroptosis in BMSCs can potentially enhance the survival of transplanted cells.

Ferritins are the only proteins capable of storing large quantities of iron, thereby playing a critical role in the regulation of cellular iron metabolism. The heavy (H)- and light (L)-chain subunits of ferritin (FTH and FTL, respectively) are responsible for intracellular iron storage [6] and recent evidence suggests that they are involved in ferroptosis and apoptosis [7, 8].

RNA-binding proteins participate in cellular metabolism and have also been implicated in ferroptosis [9]. Mitochondrial RNA-binding protein tumor necrosis factor receptor-associated protein 1 (TRAP1), also known as heat-shock protein 75-kDa, is highly expressed in a variety of cancer types and is known to induce cell death [10–12]. In particular, TRAP1-associated, chaperone-mediated autophagy can promote ferroptosis [13], while TRAP1 was shown to define a common regulatory nodal between necroptosis and ferroptosis [14]. Additionally, studies have reported that TRAP1 is an iron-binding protein associated with the plasma membrane [15]. Combined, these observations suggested that TRAP1 may also be involved in ferroptosis in BMSCs.

RNA modifications play an important role in the pathogenesis of many diseases. Methylation is a common chemical modification found in most RNAs. 5-Methylcytosine (5mC) is associated with the regulation of protein translation, RNA processing, metabolism (including iron metabolism), the stress response, and cell differentiation [16–18]. RNA-modifying enzymes that are involved in transcriptional regulation have been shown to modulate ferroptosis in stem cells. Several members of the NOP2/Sun RNA methyltransferase (Nsun) protein family have been shown to possess putative 5mC methyltransferase activity [19] and some Nsun family proteins are presumed to regulate ferroptosis in BMSCs. Nsun2 catalyzes the methylation of tRNAs and may also target mRNAs and noncoding RNAs [20]. NSUN5 is a conserved RNA (C5-cytosine) methyltransferase with a role in lifespan Modulation [21]. However, it is not known whether NSUN5 is also involved in iron metabolism.

Here, we tested the possibility that NSUN5 may mediate 5mC RNA modification in the regulation of iron metabolism and ferroptosis using BMSCs treated with erastin, a small-molecule known to induce ferroptosis. Our results provide evidence for NSUN5-mediated 5mC modification of *FTH1* and *FTL* mRNA as a novel mechanism underlying ferroptosis in BMSCs.

## Results

### Erastin-induced ferroptosis in BMSCs

**Fig. 1:**
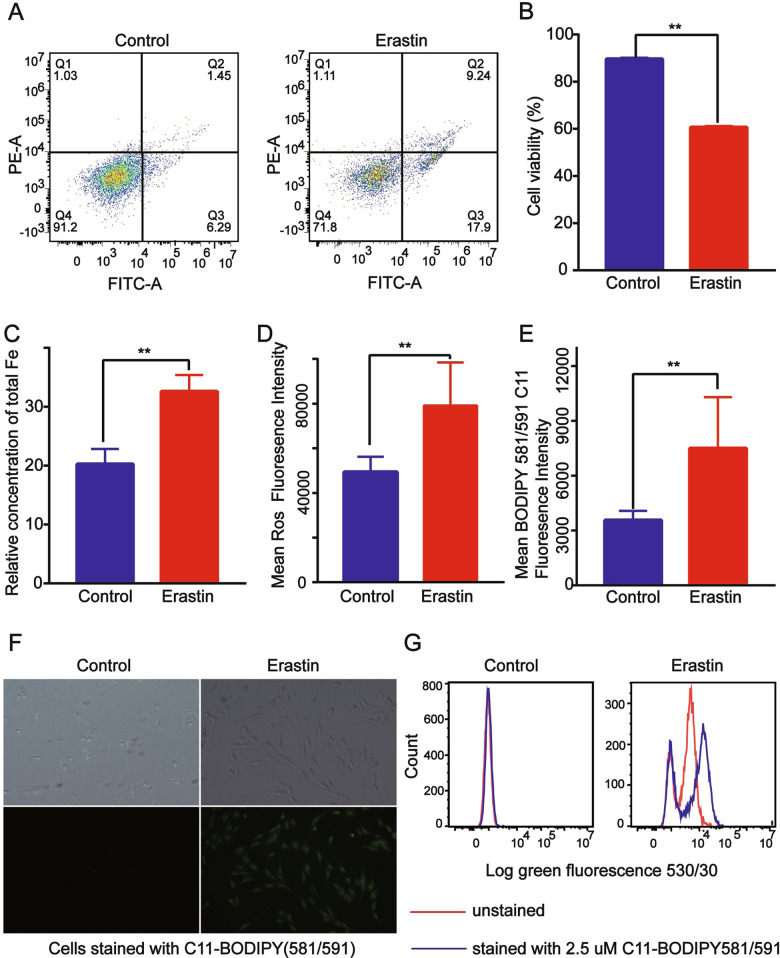
Erastin-induced ferroptosis in bone marrow-derived stem cells (BMSCs). **A–C** The fraction of dead cells (**A**, **B**) and total iron levels (**C**) were greater in BMSCs treated with erastin than in the control group. **p < 0.05. **D–G** ROS (**D**) and lipid peroxide (**E–G**) levels were higher in erastin-treated cells than in control cells. **p < 0.05 (**B–G**).

We evaluated erastin-induced BMSC ferroptosis by measuring the intracellular levels of Fe, lipid peroxidation products, and ROS. The flow cytometric analysis showed that the fraction of dead BMSCs was larger in the erastin group than in the untreated control group (Fig. 1A, B; Supplementary Fig. 1). Iron is a mediator of ferroptosis, and total iron levels were significantly higher in BMSCs treated with erastin than in control cells (Fig. 1C). ROS levels were also higher in the erastin group than in the control group (Fig. 1D). To confirm the induction of oxidative stress by erastin, we assessed lipid peroxidation in BMSCs using the C11-BODIPY 581/591 assay, and found that lipid peroxide levels were significantly higher in erastin-treated BMSCs than in control cells (Fig. 1E–G). These results suggested that erastin induces the ferroptosis of BMSCs.

### NSUN5 is downregulated in erastin-induced BMSC ferroptosis

**Fig. 2:**
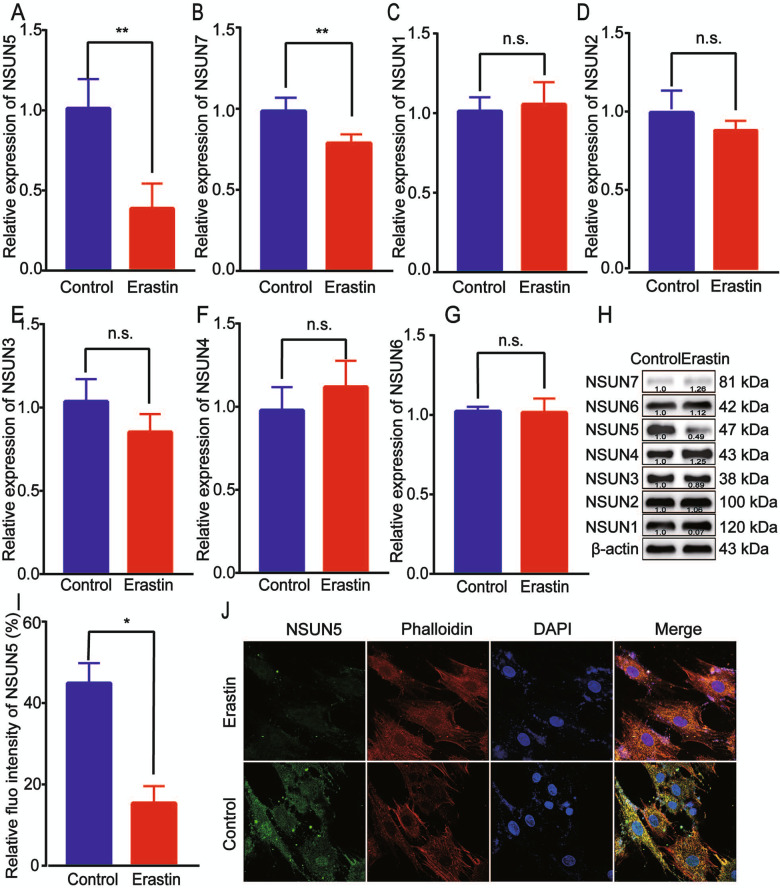
NSUN5 was downregulated in erastin-induced ferroptosis of bone marrow-derived stem cells (BMSCs). **A–H** Compared with control cells, *NSUN5* and *Nsun7* mRNA (**A–G**) and NSUN5 protein (**H**) expression levels were downregulated in erastin-induced BMSC ferroptosis as determined by RT-qPCR and western blotting, respectively. Data are presented as means ± SD. *p < 0.05. **I**, **J** Fluorescence signals associated with NSUN5 protein were lower in erastin-treated BMSCs than in control cells, *p < 0.05 (**A–J**).

We screened for the mRNAs and proteins of some RNA methyltransferases that were differentially expressed during BMSC ferroptosis using RT-qPCR and western blotting, respectively. Of the seven RNA methyltransferases evaluated, only *NSUN5* and NSUN7 mRNA expression levels differed significantly between control and erastin-treated BMSCs (Fig. 2A–H). RT-qPCR analysis showed that *NSUN5* transcript levels were downregulated by erastin treatment (Fig. 2A), and the same was true for protein levels as determined by western blotting (Fig. 2H). Immunofluorescence labeling and confocal microscopy analysis revealed that the NSUN5 fluorescence signal was weaker in erastin-treated BMSCs than in control cells (Fig. 2I, J). These results suggested that NSUN5 is downregulated in erastin-induced BMSC ferroptosis and may be a key mediator of this process.

### NSUN5 inhibits BMSC ferroptosis

**Fig. 3:**
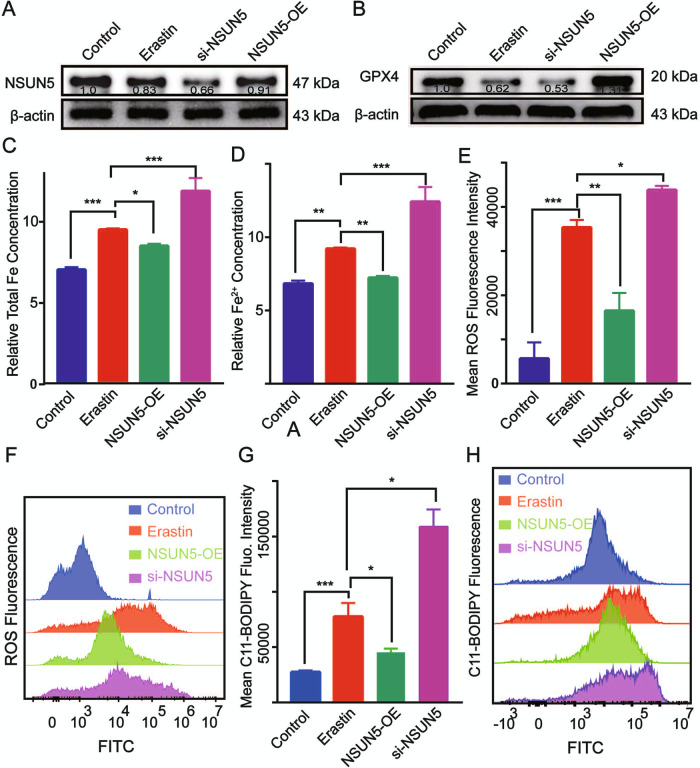
NSUN5 inhibits bone marrow-derived stem cell (BMSC) ferroptosis in vitro. **A** *NSUN5* knockdown and overexpression in BMSCs confirmed by western blotting. *p < 0.05. **B** Compared with control cells, GPX4 expression was downregulated by NSUN5 depletion in BMSCs treated with erastin. *p < 0.05. **C**, **D** Total iron concentrations (**C**) and Fe^2+^ levels (**D**) were enhanced by *NSUN5* knockdown. *p < 0.05. **E–H** Erastin-induced ROS production (**E**, **F**) and lipid peroxidation (**G**, **H**) were increased by *NSUN5* depletion and reduced by *NSUN5* overexpression, *p < 0.05 (**C–H**).

To clarify the function of NSUN5 in BMSC ferroptosis, *NSUN5* was overexpressed or silenced using lentiviral vectors. The stable knockdown or overexpression of *NSUN5* in BMSCs was confirmed by western blotting (Fig. 3A; Supplementary Fig. 2). Compared with untreated controls, the expression levels of glutathione peroxidase 4 (GPX4) were lower and higher with *NSUN5* knockdown and overexpression, respectively, in BMSCs treated with erastin (Fig. 3B). Total iron and Fe^2+^ levels were enhanced by *NSUN5* depletion (Fig. 3C, D), whereas *NSUN5* overexpression abrogated the erastin-induced increases in total iron and Fe^2+^ levels (Fig. 3C, D). ROS levels were also increased by *NSUN5* knockdown and decreased by *NSUN5* overexpression (Fig. 3E, F), and the same effect was observed for lipid peroxidation products (Fig. 3G, H). These data suggested that NSUN5 inhibits ferroptosis in BMSCs.

### FTH1 and FTL are substrates of NSUN5

**Fig. 4:**
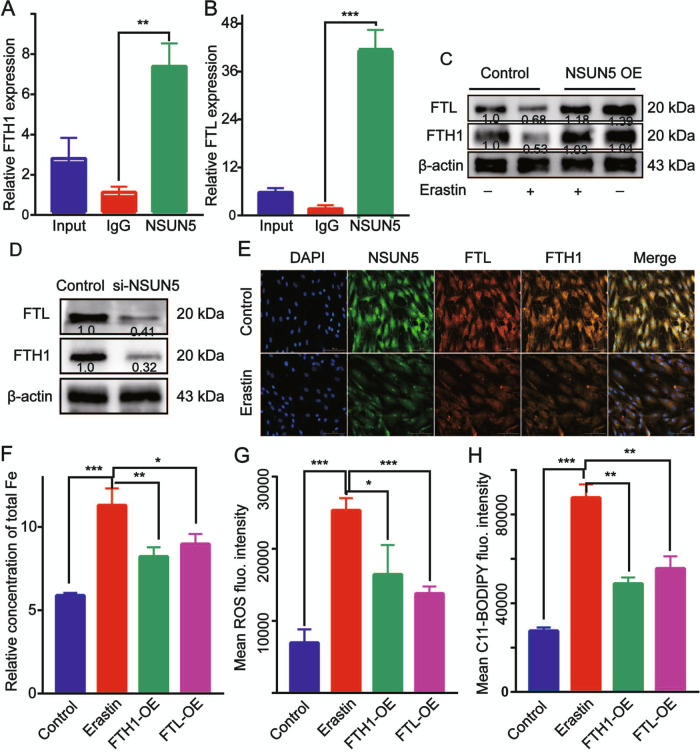
*FTH1* and *FTL* mRNA are substrates of NSUN5. **A**, **B** NSUN5 binds to *FTH1* and *FTL* mRNA in bone marrow-derived stem cells (BMSCs), as revealed by RNA immunoprecipitation (RIP), **p < 0.05. **C**, **D** NSUN5 overexpression increased the expression levels of FTH1 and FTL (**C**), whereas both ferritin subunits were downregulated by si-NSUN5 (**D**), as determined by western blotting. *p < 0.05. **E** NSUN5, *FTH1*, and *FTL* co-localized in the cytoplasm of BMSCs, as revealed by immunofluorescence analysis, *p < 0.05. **F** *FTH1* and *FTL* overexpression attenuated the erastin-induced increase in total iron concentrations, *p < 0.05. **G**, **H** Erastin-induced ROS production (**G**) and lipid peroxidation (**H**) were attenuated by *FTH1* and *FTL* overexpression in BMSCs. *p < 0.05 (**A–H**).

To clarify the mechanism by which NSUN5 inhibits ferroptosis, RIP and RT-qPCR were employed to identify its target transcripts. The results showed that NSUN5 bound *FTH1* and *FTL* mRNA in BMSCs (Fig. 4A, B). Meanwhile, western blotting analysis indicated that NSUN5 overexpression increased the expression levels of FTH1 and FTL (Fig. 4C; Supplementary Fig. 3), whereas those of both ferritin subunits were downregulated following si-NSUN5 transfection (Fig. 4D; Supplementary Fig. 3). Immunofluorescence analysis confirmed the colocalization of NSUN5, FTH1, and FTL in the cytoplasm of BMSCs (Fig. 4E; Supplementary Figs. 4, 5). These observations suggested that FTH1 and FTL are potential targets or substrates of NSUN5.

**Fig. 5:**
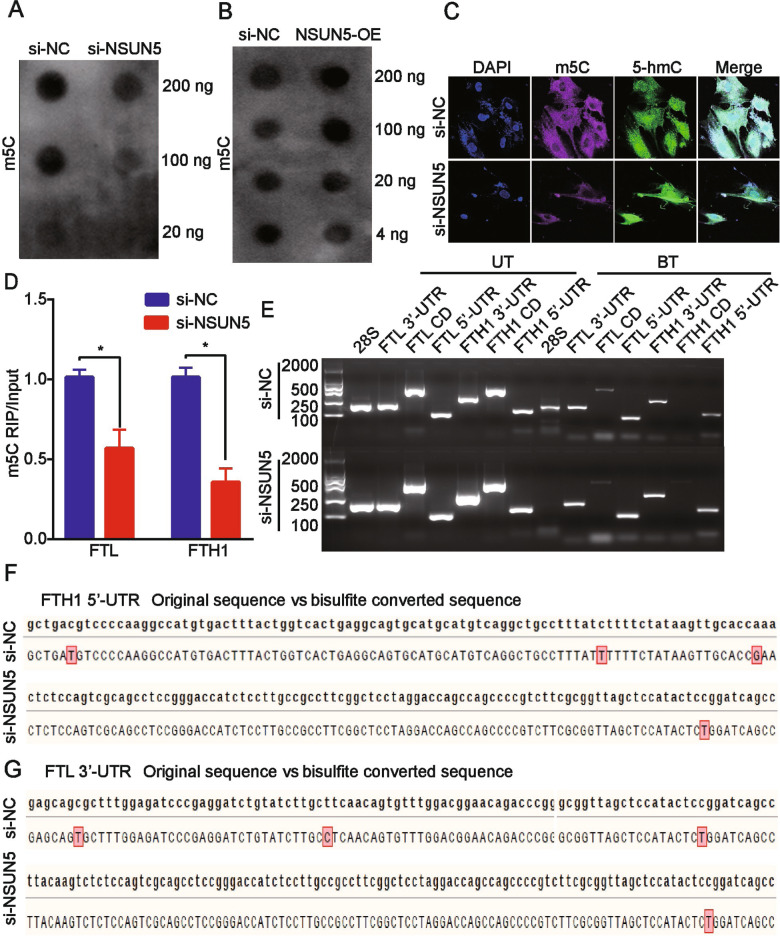
NSUN5 inhibits erastin-induced ferroptosis in bone marrow-derived stem cells (BMSCs) by 5mC modification of *FTH1* and *FTL* RNA. **A**, **B** The results of the dot blot assay showed that *NSUN5* knockdown reduced 5mC levels, whereas *NSUN5* overexpression elicited the opposite effect. *p < 0.05. **C** Compared with control cells, 5mC levels were reduced in *NSUN5*-depleted cells, as determined by immunofluorescence analysis. *p < 0.05. **D** 5mC antibody enriched *FTH1* and *FTL* mRNA, whereas *NSUN5* knockdown reduced 5mC levels in *FTH1* and *FTL* mRNA, as determined by RNA immunoprecipitation (RIP) and reverse transcription-quantitative polymerase chain reaction (RT-qPCR) analysis. *p < 0.05. **E** Methylation sites of *FTH1* and *FTL* mRNA in control of *NSUN5*-depleted BMSCs were mainly in the 5′ or 3′ UTR. **F**, **G** Bisulfite-sequencing analysis revealed that *NSUN5* knockdown abolished the C-to-T conversion at *FTH1* and *FTL* mRNA cytosine methylation sites, *p < 0.05 (**D**).

To determine whether FTH1 and FTL mediate the inhibitory effect of NSUN5 on BMSC ferroptosis, we overexpressed *FTH1* and *FTL* in erastin-treated BMSCs and found that the increase in total iron concentration induced by erastin was abolished (Fig. 4F). This was accompanied by a decrease in ROS levels (Fig. 4G) and the partial abrogation of lipid peroxidation (Fig. 4H), both of which were induced by erastin. The results of the dot blot assay showed that *NSUN5* knockdown reduced 5mC levels, whereas *NSUN5* overexpression had the opposite effect (Fig. 5A, B; Supplementary Fig. 6). Immunofluorescence analysis of 5mC levels using confocal microscopy revealed that 5mC-associated fluorescence was weaker in *NSUN5*-depleted BMSCs than in control cells (Fig. 5C).

We next evaluated the functional significance of the NSUN5-mediated 5mC modification of *FTH1* and *FTL* mRNA. The results of the 5mC RIP assay and RT-qPCR showed that the anti-5mC antibody enriched the mRNA of *FTH1* and *FTL*. *NSUN5* silencing reduced the 5mC levels in both transcripts, suggesting that NSUN5 regulates *FTH1* and *FTL* expression *via* 5mC RNA modification (Fig. 5D).

We also assessed the methylation status of *FTH1* and *FTL* mRNAs in BMSCs by bisulfite sequencing using *FTH1*- and *FTL*-specific primers and found that they were methylated in the 5′ (FTH1) and 3′ (FTL) untranslated regions (UTRs) (Fig. 5E–G). The nucleotide was the 5mC methylation site for FTH1, and the nucleotide 628 was the 5mC methylation site for FTL (Fig. 5F, G). Sequence analysis indicated that *NSUN5* depletion induced the C-to-T conversion at *FTH1* and *FTL* mRNA cytosine methylation sites, lead to the reduce of the methylate modification. These results suggested that 5mC levels in *FTH1* and *FTL* RNA are decreased with NSUN5 knockdown, leading to a reduction in FTH1 and FTL protein synthesis.

### NSUN5 recruits TRAP1 to modify FTH1 and FTL

**Fig. 6:**
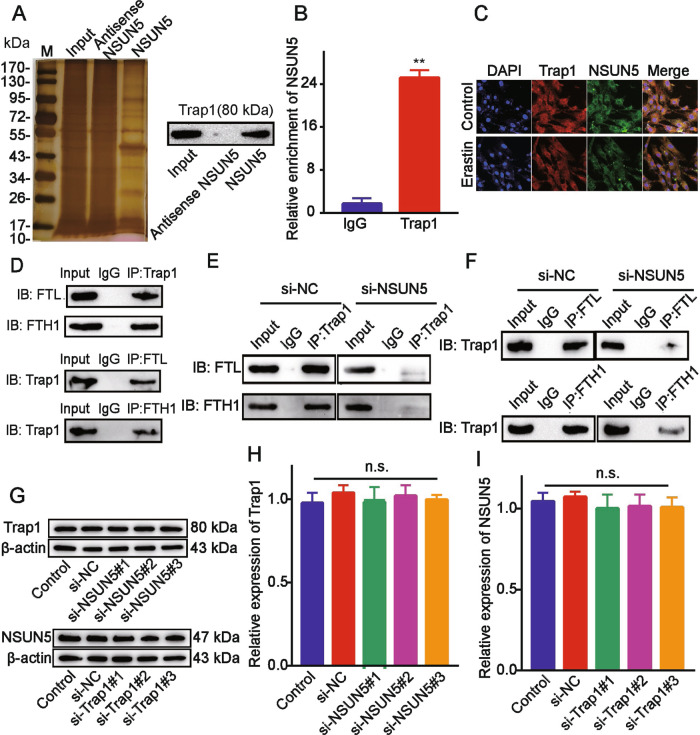
NSUN5 recruits TRAP1 to modify *FTH1* and *FTL*. **A** Silver-stained gel showing a protein band among the pulldown products of bone marrow-derived stem cells (BMSCs) expressing *NSUN5* that was absent in those of *NSUN5*-depleted cells; western blot analysis confirmed TRAP1 enrichment in the former group. **B** Detection of NSUN5/TRAP1 binding complexes by RNA immunoprecipitation (RIP). *p < 0.05. **C** Immunofluorescence analysis confirmed that NSUN5 and TRAP1 protein co-localized in the cytoplasm of BMSCs. *p < 0.05. **D** Interaction between TRAP1 and *FTH1*/*FTL* revealed by co-immunoprecipitation (co-IP). **E**, **F** *NSUN5* knockdown reduced the amount of precipitated *FTH1*/*FTL*, indicating that the interaction between TRAP1 and *FTH1*/*FTL* was abolished. **G–I** *NSUN5* silencing did not affect the mRNA and protein levels of TRAP1 and vice versa, *p < 0.05 (**B**).

**Fig. 7:**
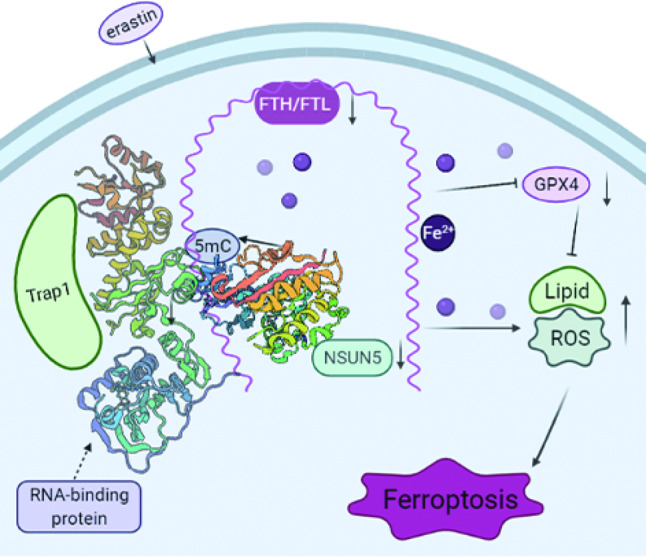
Schematic diagram showing the mechanisms of NSUN5-FTH1/FTL. After added erastin, NSUN5 methylated FTH1/FTL mRNA by targeting on its 5′UTR/3′UTR region. NSUN5 recruits trap1 and binds to FTH1/FTL mRNA in BMSCs, and then induced to the degrade of FTH1/FTL. After FTH1/FTL is downregulated, it further promotes the low level of iron storage. Eventually, FTH1/FTL is maintained at an extremely low level, resulting in the accumulation of free ferrous ions, ROS levels, lipid peroxidation products and a reduction in the GPX4 and eventually leading to cell death due to ferroptosis.

We investigated whether other proteins are involved in the NSUN5-mediated modification of *FTH1* and *FTL*. Using silver staining and western blotting, we found a protein band among the pulldown products of BMSCs expressing NSUN5 that was absent in those of NSUN5-depleted cells (Fig. 6A), which was identified as TRAP1. The same result was obtained in the LC–MS analysis (Supplementary Materials LC–MS report). The specific enrichment of TRAP1 in lysates of NSUN5-expressing BMSCs was confirmed by western blotting, and vice versa (Fig. 6A, Supplementary Fig. 7). TRAP1 is a mitochondrial paralog of heat-shock protein 90 (Hsp90) that has been reported to induce cell death and may also regulate ferroptosis in BMSCs. We speculated that NSUN5 might regulate *FTH1* and *FTL* through the recruitment of TRAP1. RIP analysis identified an abundance of NSUN5–TRAP1 complexes (Fig. 6B), while*L* through the recruitment oimmunofluorescence analysis showed that NSUN5 and TRAP1 proteins co-localized in the cytoplasm*L* through the recruitment oof BMSCs (Fig. 6C; Supplementary Fig. 8). The interaction between TRAP1 and *FTH1* and *FTLL* through the recruitment owas confirmed by a co-IP assay (Fig. 6D). To examine the role of NSUN5 in the TRAP1-*FTH1*/*FTLL* through the recruitment ointeraction, we silenced *NSUN5* expression in BMSCs, and found that the amount of *FTH1*/*FTLL* through the recruitment oprecipitated in TRAP1 complexes was reduced. This suggested that, in the absence of NSUN5, the*L* through the recruitment ointeraction between TRAP1 and *FTH1*/*FTL* was abolished (Fig. 6E, F). In contrast, NSUN5 knockdown had no effect on the protein (Fig. 6G) or mRNA (Fig. 6H, I) levels of TRAP1 and vice versa. These findings suggested that NSUN5 recruits TRAP1 to modify *FTH1* and *FTL* (Fig. 7; Supplementary Fig. 9).

## Discussion

MSC transplantation is used for the treatment of several diseases; however, the ferroptosis of BMSCs can lead to transplant failure, thereby limiting their use. Consequently, it is important to elucidate the mechanisms underlying ferroptosis so as to identify ways of preventing its occurrence and improving the success rate of transplantations. Here, we used erastin treatment to investigate the roles of NSUN5 in BMSC ferroptosis. The major findings of the present study were as follows: (1) NSUN5 was downregulated in erastin-induced BMSC ferroptosis, while NSUN5 overexpression elicited the opposite effects; (2) *FTH1* and *FTL* are substrates of NSUN5; and (3) NSUN5 recruits TRAP1 to modify FTH1 and FTL. These findings suggest that the impairment of the NSUN5-FTH1/FTL pathway could be a causative event leading to the ferroptosis of BMSCs, and that the NSUN5-FTH1/FTL pathway might be a key target for the development of drugs that can protect against ferroptosis in transplanted BMSCs.

In this study, we used erastin to induce ferroptosis in BMSCs. Erastin functions as a small-molecule inducer of ferroptosis by inhibiting GPX4, which leads to the depletion of glutathione and, eventually, cell death [22–24]. Erastin has been shown to induce ferroptosis in both SH-SY5Y and tumor cells [25, 26]. NSUN5 was found to be downregulated in erastin-treated BMSCs. Ferroptosis was alleviated by *NSUN5* overexpression and was exacerbated by *NSUN5* knockdown, suggesting that RNA methyltransferase activity regulates this process. Some Nsun proteins are presumed to regulate BMSC ferroptosis. NSUN5 is a conserved RNA (5C-cytosine) methyltransferase with a role in the modulation of longevity and cell death [21]. Considering the function of NSUN5 and its potential downstream mechanisms, investigations into the role of NSUN5 in BMSC ferroptosis may help to reveal the molecular basis underlying NSUN5 functions in BMSCs and potentially improve the survival of transplanted BMSCs.

Ferroptosis is believed to be related to the regulation of lysosomal function, glutamate metabolism, the transsulfuration pathway, and iron overload [24, 27–29]. Dysregulation of iron and redox homeostasis, autophagic degradation, and iron metabolism barriers also play important roles in the mechanism of ferroptosis [30, 31]. Iron overload affects ferritin deposition and systemic iron homeostasis [32, 33]. High concentrations of iron can lead to the overproduction and aggregation of iron-containing ferritin, which has been observed in the liver and spleen [34]. Ferritin comprises heavy- and light-chain subunits, FTH and FTL, which are responsible for intracellular iron storage [6], play important roles in cell death, and may also be involved in ferroptosis [35, 36]. In our study, *FTH1* and *FTL* levels—which were positively correlated with those of NSUN5—were reduced in erastin-induced ferroptosis, and this was associated with increased intracellular iron concentrations in BMSCs. Conversely, ferroptosis was attenuated by *FTH1*/*FTL* overexpression. We further determined that NSUN5 binds to *FTH1*/*FTL* mRNA and that the latter are targets for the RNA methyltransferase activity of NSUN5. Interestingly, *FTH1* underwent 5mC modification in its 5′ UTR, whereas that of *FTL* occurred in the 3′ UTR. Nevertheless, the functional significance of this observation remains to be determined.

We identified TRAP1 as an interaction partner of NSUN5 in BMSC cells. The co-IP assay showed that NSUN5 recruits TRAP1 to bind to *FTH1* and *FTL* mRNA. Taken together, our findings suggest a mechanism in which the downregulation of NSUN5 leads to reduced 5mC levels in *FTH1*/*FTL* mRNA and a consequent increase in intracellular iron concentrations, decreased GPX4 levels and activity, and accumulation of ROS and lipid peroxidation products, all of which promote ferroptosis.

Our findings have several important clinical implications. First, the treatment of acute liver failure, neurodegenerative diseases, and numerous other conditions are limited by the low survival rate of transplanted BMSCs [37], highlighting that methods to improve survival rates are urgently needed. We found that NSUN5 was downregulated in erastin-induced BMSC ferroptosis and that its overexpression inhibited BMSC ferroptosis. These observations indicate that NSUN5 may be a potential biomarker for the status of BMSCs, and could be used to improve the survival rate of transplanted BMSCs. Second, our results support that the regulation of the NSUN5-FTH1/FTL pathway represents a novel putative therapeutic target for the prevention of ferroptosis in transplanted BMSCs.

In conclusion, our study is the first to demonstrate that NSUN5 suppresses ferroptosis in BMSCs through interaction with TRAP1 and 5mC modification of *FTH1* and *FTL* mRNA. The therapeutic targeting of NSUN5-FTH1/FTL pathway components has the potential to improve the survival of transplanted BMSCs.

## Materials and methods

### Extraction and culture of BMSCs

Sprague Dawley rats (4 weeks old) were obtained from the Animal Center of Guangzhou University of Chinese Medicine and were housed in standard laboratory rooms under controlled conditions. All animal experiments were performed according to international guidelines for the care and use of laboratory animals and were approved by the Experimental Animal Committee of Guangzhou University of Chinese Medicine. BMSCs were extracted and cultured according to our previously described protocol^24^. Briefly, bone marrow plugs were extracted from the bones of the hind legs of the rats, and the cells were centrifuged and resuspended twice in complete medium (Cyagen Biosciences, Santa Clara, CA, USA). Subsequently, 5 × l0^8^ cells in 9 mL of complete medium were transferred to 100-mm culture dishes. Three days later, the medium was refreshed and only adherent cells were maintained in complete medium supplemented with glucose and 10% (*v/v*) fetal bovine serum (Invitrogen, Carlsbad, CA, USA) until treatment with erastin (MedChemExpress, Monmouth Junction, NJ, USA).

### Lentivirus overexpression and knockdown experiments

A lentiviral vector (lenti dCAS-VP64) harboring the green fluorescence protein (GFP) coding sequence was used to stably express *NSUN5* in BMSCs. We obtained Nsun5 from a different vector and then inserted the sequence into the lenti dCAS-VP64 vector along with the GFP from pLVX-AcGFP, along with one containing a scrambled sequence as a negative control for the overexpression experiments (lenti-dCas9-Vp64-AcGFP). A total of 5 × 10^5^ cells/well were seeded in 6-well plates and incubated for 12 h. At 50% confluence, the cells were infected with either lenti-dCas9-Vp64-NSUN5-AcGFP (NSUN5 overexpression group) or lenti-dCas9-Vp64-AcGFP (negative control group), or not infected. The cells were incubated for 24 h before the medium was replaced with virus-free medium. Three days later, the infection efficiency was evaluated based on GFP signal intensity, and the cells were harvested for western blot and real-time quantitative PCR (RT-qPCR) analyses. For the knockdown experiments, BMSCs were transfected with siRNAs targeting NSUN5 and TRAP1. The siRNAs were designed and synthesized by RiboBio (Guangzhou, China).

### C11-BODIPY 581/591 assay

The C11-BODIPY assay was used to characterize the degree of lipid peroxidation, as previously described [38, 39]. BMSCs were first incubated in basal medium without (control) or with erastin (20 μM) for 12 h and then with C11-BODIPY (2.5 μM, Molecular Probes, Carlsbad, CA, USA) for 30 min. The cells were observed and imaged under a fluorescence microscope (Olympus) or analyzed using flow cytometry (Beckman Coulter, CA, USA).

### Measurement of intracellular iron concentrations

To measure intracellular iron concentrations, BMSCs were seeded at 1 × 10^4^ cells/well in 96-well plates. After allowing them to adhere for 24 h, the cells were divided into groups as in the C11-BODIPY assay. The iron concentration was quantified using the QuantiChrom Iron Assay Kit (Bioassay Systems, Hayward, CA, USA), and the reduction of Fe^3+^ to Fe^2+^ in the presence of the chromogen was quantified by measuring the absorbance at 590 nm using a spectrophotometer (BioTek, VT, USA).

### Measurement of ROS levels

To measure ROS production, BMSCs were seeded at 1 × 10^6^ cells/well in 12-well plates. After allowing them to adhere for 24 h, the cells were divided into groups as in the C11-BODIPY assay. The cells were incubated with 10 μM DCF-DA (Molecular Probes) and ROS levels were determined using flow cytometry (Beckman Coulter).

### RT-qPCR

*NSUN5* transcript levels were evaluated using RT-qPCR. Total RNA was extracted from BMSCs using TRIzol (Invitrogen). RNA purity and concentration were determined by measuring the absorbance at 280 and 260 nm using a NanoDrop-2000 spectrophotometer (Thermo Fisher Scientific, Waltham, MA, USA). The RNA was converted into cDNA using the RevertAid First Strand cDNA Synthesis Kit (Thermo Fisher Scientific) according to the manufacturer’s instructions. Primers for amplifying *NSUN5*, *Trap1*, *Fth1*, *Ftl*, and beta-actin were obtained from Sangon Biotech (Shanghai, China). The sequences of the primers used are shown in Supplementary Table 1. qPCR was performed in a LightCycler 480 II instrument (Roche Applied Science, Basel, Switzerland) using a SYBR Green Master Mix.

### Western blot

Proteins were extracted from BMSCs using RIPA lysis buffer containing a protease inhibitor cocktail (Sigma, MO, USA). Proteins (30 µg) were separated on precast 10% SDS–polyacrylamide gels (Bio-Rad, USA) and then transferred to nitrocellulose membranes (Millipore Sigma, USA) by semi-dry blotting. After blocking with 5% skim milk, the cells were incubated for 12 h at 4 °C with primary antibodies targeting NSUN5 (Proteintech, IL, USA), FTH1, FTL, β-actin, 5mC (Abcam, Cambridge, MA, USA), or DDX25 (Santa Cruz, CA, USA). The next day, the membranes were incubated with an horseradish peroxidase (HRP)-conjugated secondary antibody. Protein bands were visualized by enhanced chemiluminescence (Millipore, MA, USA).

### Fluorescence immunocytochemistry

BMSCs were fixed in 4% formalin, washed in phosphate-buffered saline, and then permeabilized with 0.25% Triton X-100. Nonspecific binding was blocked using serum-supplemented antibody dilution buffer for 30 min. Immunofluorescence labeling was performed using monoclonal antibodies against NSUN5, TRAP1, FTH1, and FTL diluted 1:100 in 5% goat serum. After incubation for 12 h, the cells were rinsed and incubated for 1 h at room temperature with fluorochrome-conjugated secondary antibodies (Alexa Fluor 488, Alexa Fluor 555, Alexa Fluor 594, or Alexa Fluor 647; Abcam). After counterstaining with DAPI and washing, the cells were imaged using a confocal microscope.

### RNA immunoprecipitation assay

RNA immunoprecipitation (RIP) was performed using the Magna RIP RNA-Binding Protein Immunoprecipitation Kit (Merck Millipore, Billerica, MA, USA) according to the manufacturer’s protocol. Briefly, cultured BMSCs were collected and lysed in RIP lysis buffer. Thewhole-cell extracts were incubated with RIP buffer containing magnetic beads cross-linked with antibodies targeting NSUN5, 5mC, or control IgG at 4 °C for 8 h. The beads were washed and the complexes were incubated with proteinase K and SDS at 55 °C for 30 min to remove proteins. Co-precipitated RNAs were amplified using RT-qPCR.

### Dot blot assay

RNA was diluted to 200, 100, 50, and 4 ng/µL. Nitrocellulose membranes were predipped in methyl alcohol and a dot blot apparatus was used to spot the RNA onto the membranes. Approximately 200, 100, 50, and 4 ng of RNA was probed to measure 5mC modification levels. The RNA was cross-linked to the membranes using ultraviolet irradiation and the membranes were incubated with blocking solution, followed by an anti-5mC antibody diluted in blocking buffer, and then an HRP-conjugated secondary antibody. The blots were visualized using enhanced chemiluminescence.

### Methylation analysis by bisulfite sequencing

Total RNA was extracted from BMSCs and purified using an RNA extraction kit (Zymo Research, Irvine, CA, USA) according to the manufacturer’s instructions. The RNA was treated with bisulfite and PCR was performed under the following conditions: predenaturation at 95 °C for 10 min, followed by 40 cycles of 95 °C for 30 s, 60 °C for 40 s, and 72 °C for 40 s, with a final extension at 72 °C for 10 min. The methylation status of the *Fth1* and *Ftl* genes was qualitatively analyzed using gel electrophoresis (1× TAE) and methylation-specific PCR, gene sequencing, and direct bisulfite sequencing. The primer sequences are listed in Supplementary Table 2.

### Co-immunoprecipitation assay

Co-immunoprecipitation (co-IP) was performed using the Magna RIP RNA-Binding Protein Immunoprecipitation Kit (Thermo Fisher Scientific) according to the manufacturer’s protocol. Briefly, BMSCs were collected from culture plates and lysed in co-IP lysis buffer. The lysates were centrifuged at 13,000 × *g* at 4 °C for 15 min. The extracted proteins were incubated at 4 °C for 12 h with magnetic beads cross-linked with nonspecific mouse IgG (5 mg) or anti-NSUN5/Trap1 antibodies and treated with coupling buffer. After magnetic separation, the precipitates were washed, the proteins were eluted and neutralized, and western blotting and silver staining were performed according to standard protocols.

### Silver staining assay and liquid chromatography–mass spectrometric analysis

Co-IP products were analyzed using vertical gel electrophoresis on 12.5% polyacrylamide nondenaturing gels at 300 V for 45 min. The gels were subjected to silver staining and sent to BGI Technology Co., Ltd for analysis by liquid chromatography–tandem mass spectrometry (LC–MS).

### Statistical analysis

Each experiment was performed in triplicate. Data were expressed as means ± standard deviation and were analyzed using GraphPad Prism v7.0 (GraphPad Software, La Jolla, CA, USA) or SPSS v19.0 (SPSS Inc, Chicago, IL, USA). The Student’s *t* test was used to compare means between two groups and a *p* value of <0.05 was considered statistically significant.

## Supplementary information

Supplementary material

Supplementary Table 1

Supplementary Table 2

Supplementary Figure 1

Supplementary Figure 2

Supplementary Figure 3

Supplementary Figure 4

Supplementary Figure 5

Supplementary Figure 6

Supplementary Figure 7

Supplementary Figure 8

Supplementary Figure 9

## Data Availability

The datasets used and analyzed during the current study are available from the corresponding author on reasonable request.
