# Supplementary material for: The NSUN5-FTH1/FTL pathway mediates ferroptosis in bone marrow-derived mesenchymal stem cells
Source: Cell Death Discov. 2022 Mar 5;8:99. doi: 10.1038/s41420-022-00902-z (PMC8898311; doi:10.1038/s41420-022-00902-z)
Supplement: Supplementary file 1 — Supplementary material [file 41420_2022_902_MOESM1_ESM.pdf]

Identification Proteomics Report

2019/5/22

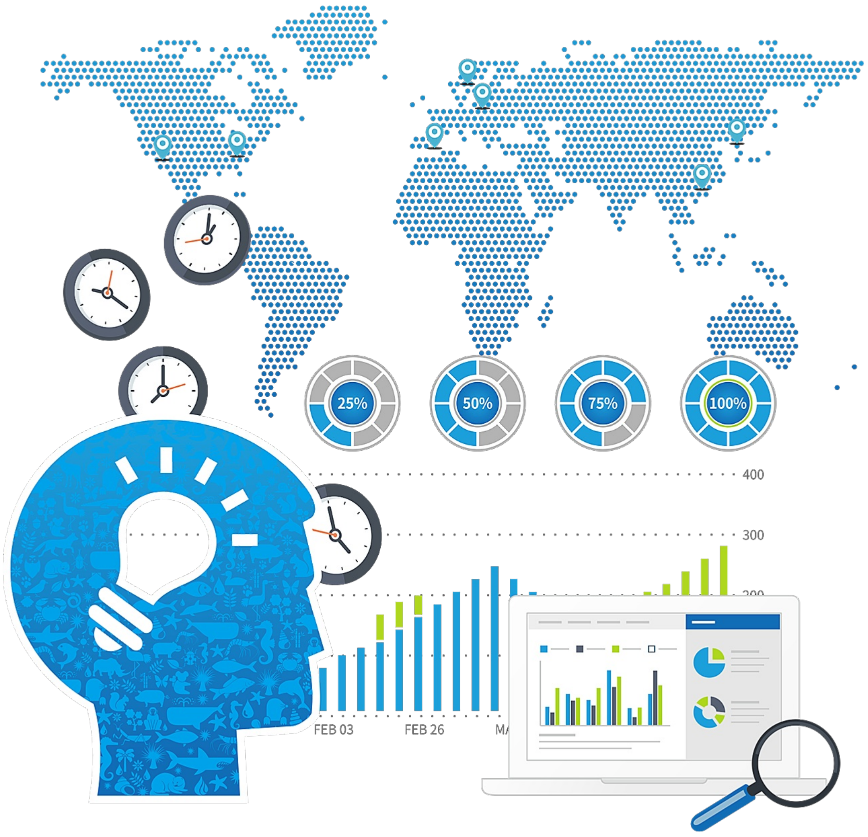

@2019 BGI All Rights Reserved

目 录

分析结果 2

1 蛋白鉴定结果 2

2 功能分析 8

分析方法 10

1 实验流程 10

2 信息分析流程 12

帮助 14

1 mgf数据格式说明 14

2 数据目录编排说明 14

3 蛋白质序列FASTA格式 15

4 peptideSummary文件格式说明 15

5 proteinSummary文件格式说明 16

6 GO功能注释说明 16

7 COG功能注释说明 17

8 Pathway通路注释说明 18

常见问题 19

参考文献 19

 © 2017 BGI All Rights Reserved.

●分析结果

1 蛋白鉴定结果

1.1 概述

本项目所用质谱仪器是 Triple TOF 5600。样品组nsun5_co-ip_control中共得到谱图31265张，通过搜索引擎完成鉴定后，匹配到的谱图数量是3904张，共鉴定到446个蛋白，1228个肽段。样品组n5_co-iperastin_erastin中共得到谱图28295张，通过搜索引擎完成鉴定后，匹配到的谱图数量是884张，共鉴定到133个蛋白，337个肽段。

表1 鉴定结果概览 （下载）

Sample	Total_spectra	Identified_spectra	Identified_peptides	Identified_proteins
nsun5_co-ip_control	31265	3904	1228	446
n5_co-iperastin_erastin	28295	884	337	133

1.2 基本统计图

下面为蛋白鉴定结果的基本统计图，分别从“唯一谱图分布”，“唯一肽段分布”，“蛋白质量分布”，“蛋白覆盖度分布”以及“肽段长度分布”五个方面分别进行绘制。

搜索 显示

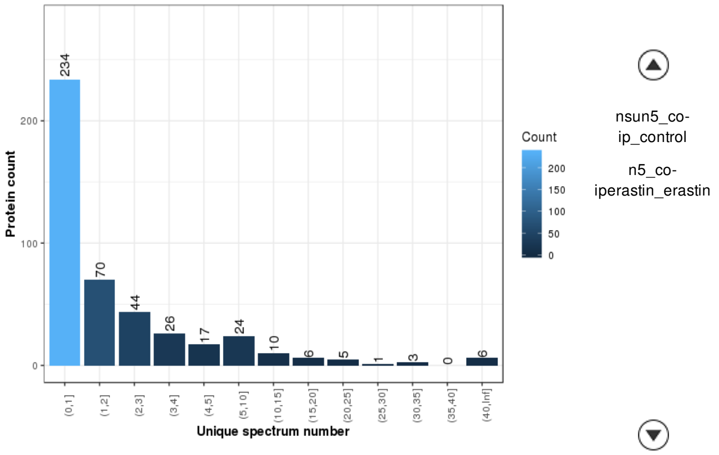

图1 唯一谱图分布.。

横轴为每个蛋白的唯一匹配谱图数，纵轴为蛋白数目

深圳华大基因股份有限公司 400-706-6615 © 2017 BGI All Rights Reserved.

搜索 显示

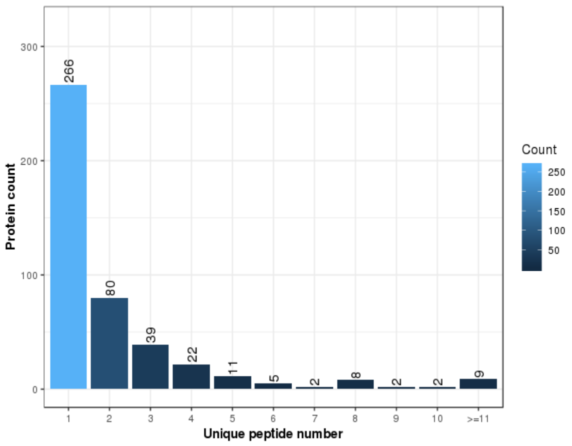

nsun5_co-ip_control

n5_co-iperastin_erastin

图2 唯一肽段分布。

横轴为每个蛋白的唯一匹配肽段数，纵轴为蛋白数目

搜索 显示

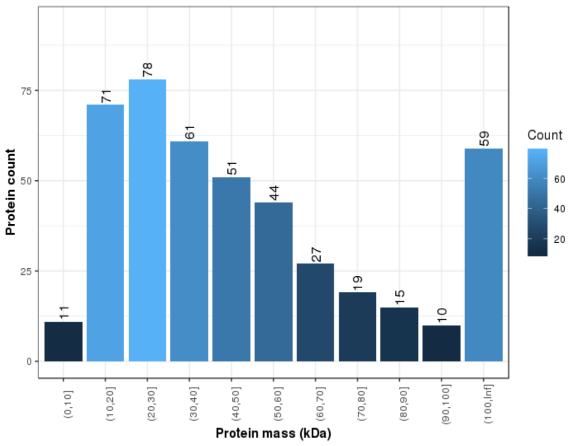

nsun5_co-ip_control

n5_co-iperastin_erastin

图3 蛋白质量分布。

横轴为蛋白质量区间（Kilodalton），纵轴为相应蛋白数目

© 2017 BGI All Rights Reserved.

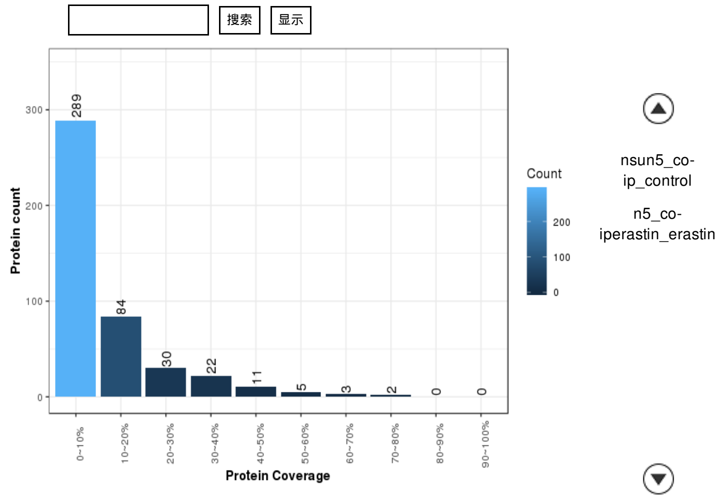

图4 蛋白覆盖度分布。

横轴为蛋白覆盖度百分比区间，纵轴为蛋白数目

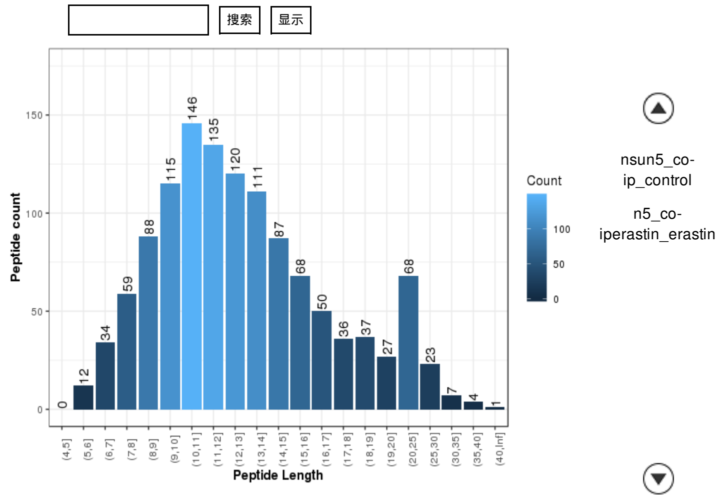

图5 肽段长度分布。

横轴为每个肽段的长度区间（残基个数），纵轴为相应肽段数目

1.3 详细鉴定列表

以下为各样品“谱图鉴定列表”,“肽段鉴定列表”和“蛋白鉴定列表”，每个列表只预览前10行。完整列表可通过各列表的“*查看全部*”按钮进行查看。

表2 谱图鉴定列表:nsun5_co-ip_control （查看全部）

深圳华大基因股份有限公司 400-706-6615 © 2017 BGI All Rights Reserved.

query_number	peptide_seq	PSM_FDR	ions_score	Protein_ID
29687	TTGIVMDSGDGVTHTVPIYEGYALPHAILR	0	4.41629	sp|P60711|ACTB_RAT;sp|P63259|A
29691	TTGIVMDSGDGVTHTVPIYEGYALPHAILR	0	4.20809	sp|P60711|ACTB_RAT;sp|P63259|A
29688	TTGIVMDSGDGVTHTVPIYEGYALPHAILR	0	4.20223	sp|P60711|ACTB_RAT;sp|P63259|A
29789	TTGIVMDSGDGVTHTVPIYEGYALPHAILR	0	4.19918	sp|P60711|ACTB_RAT;sp|P63259|A
29790	TTGIVMDSGDGVTHTVPIYEGYALPHAILR	0	4.18953	sp|P60711|ACTB_RAT;sp|P63259|A
29698	TTGIVMDSGDGVTHTVPIYEGYALPHAILR	0	4.03837	sp|P60711|ACTB_RAT;sp|P63259|A
29686	TTGIVMDSGDGVTHTVPIYEGYALPHAILR	0	3.95992	sp|P60711|ACTB_RAT;sp|P63259|A
29680	TTGIVMDSGDGVTHTVPIYEGYALPHAILR	0	3.72921	sp|P60711|ACTB_RAT;sp|P63259|A
29697	TTGIVMDSGDGVTHTVPIYEGYALPHAILR	0	3.67943	sp|P60711|ACTB_RAT;sp|P63259|A
29685	TTGIVMDSGDGVTHTVPIYEGYALPHAILR	0	3.6555	sp|P60711|ACTB_RAT;sp|P63259|A

表3 谱图鉴定列表:n5_co-iperastin_erastin （查看全部)

query_number	peptide_seq	PSM_FDR	ions_score	Protein_ID
27151	TTGIVMDSGDGVTHTVPIYEGYALPHAILR	0	3.64919	sp|P60711|ACTB_RAT;sp|P63259|A
18953	LQQELDDLLVDLDHQR	0	3.41272	sp|Q62812|MYH9_RAT;tr|G3V6P7|
26387	GIYETPAGTILYHAHLDIEAFTMDR	0	3.08284	sp|P09034|ASSY_RAT
27152	TTGIVMDSGDGVTHTVPIYEGYALPHAILR	0	2.98041	sp|P60711|ACTB_RAT;sp|P63259|A
22873	QPYAVSELAGHQTSAESWGTGR	0	2.85765	sp|P50878|RL4_RAT;tr|Q6P3V9|Q6
11880	QEYDESGPSIVHR	0	2.8465	sp|P60711|ACTB_RAT;sp|P63259|A
15049	NFINNPLAQADWAAK	0	2.71754	tr|G3V6P7|G3V6P7_RAT
18952	LQQELDDLLVDLDHQR	0	2.70664	sp|Q62812|MYH9_RAT;tr|G3V6P7|
18197	VLDFEHFLPMLQTVAK	0	2.56592	sp|Q64119|MYL6_RAT;tr|A0A0G2J
21158	FHSDWCSPPNCDEIHDR	0	2.39667	sp|Q99MU5|SPAT6_RAT;tr|B0BMV

表4 肽段鉴定列表:nsun5_co-ip_control （查看全部)

Peptide	Modification	Query
ALELDSNLYR	-	4840;4838;4839;4835;4821;4822
IAQLEEELEEEQSNMELLNDR	Oxidation (M)@15M	27201
YAFVNWINK	-	3982
YLTVAAVFR	-	2337;2335
LENEIQTYR	-	4223;4224
HQGVMVGMGQK	-	4341;4342;4339;4333;4337;4325;4332;4340;180;4324;4336;4331;4335;4343;43
IEIAQK	Deamidated (NQ)@5Q	5
GWDENVYYTVPLVR	-	16280
LLLNNDNLLR	Deamidated (NQ)@4N	4926
LAAMATLLAR	-	2220

© 2017 BGI All Rights Reserved.

表5 肽段鉴定列表:n5_co-iperastin_erastin （查看全部)

Peptide	Modification	Query	Group_ID	Group_Prin
ATDAEADVASLNR	-	7876	15_4	tr|A0A0G2K
LENEIQTYR	-	4513	14_7	tr|A0A0G2K
LAAMATLLAR	-	2439	70_1	tr|G3V660|G
AGFAGDDAPR	-	1559	36_4	sp|P63259|A
TTGIVMDSGDGVTHTVPIYEGYALPHAILR	Oxidation (M)@6M	27191;27192;27190;27193;27194	36_4	sp|P63259|A
IIGLDQVAGMSETALPGAFK	Oxidation (M)@10M	19994	8_2	tr|G3V6P7|C
DETVSDCSPHIANIGR	Carbamidomethyl (C)@7C	16688	84_1	tr|A0A0G2J
GQTGGDVNVEMDAAPGVDLSR	Deamidated (NQ)@8N	20497	14_1	tr|A0A0G2JI
LISWYDNEYGYSNR	Deamidated (NQ)@7N	16782	3_1	tr|M0R590|M
ELAPYDENWFYTR	-	15551	50_1	tr|A0A0H2U

表6 蛋白鉴定列表:nsun5_co-ip_control （查看全部)

Group_ID	Protein_ID	Protein_Qscore	Protein_FDR	Protein_Mass	PeptideSeqs
327_1	sp|P35427|RL13A_RAT	4.52507894392	NA	23461.3867047	YQAVTATLEEK
332_1	sp|Q4KM74|SC22B_RAT	2.63020205857	NA	24724.7498547	NLGSINTELQDVQ
381_1	sp|P62836|RAP1A_RAT	4.52507894392	NA	20973.7076847	INVNEIFYDLVR
329_1	sp|P42123|LDHB_RAT	6.80351988031	NA	36589.0651547	FIIPQIVK;SLADELA
229_1	sp|P45592|COF1_RAT	7.2188709248	NA	18520.6679947	EILVGDVGQTVDD

深圳华大基因股份有限公司 400-706-6615 © 2017 BGI All Rights Reserved.

347_1	tr|B2RYW7|B2RYW7_RAT	2.00358778917	NA	12501.7694247	VLLESEQFLTELTF
387_1	sp|P04762|CATA_RAT	2.16737496737	NA	59719.4848147001	EAETFPFNPFDLTI
136_1	tr|M0R979|M0R979_RAT	2.63020205857	NA	126960.446044701	QVTQSYWDTNPT
330_1	tr|D4A5I9|D4A5I9_RAT	14.6807109613	NA	148037.6646847	IVEANPLLEAFGN/
345_1	tr|A0A0G2K0Y0|A0A0G2K0Y0_RAT	2.1769962896	NA	93508.5790547005	EGTAMLLEHLAGS

表7 蛋白鉴定列表: n5_co-iperastin_erastin （查看全部）

Group_ID	Protein_ID	Protein_Qscore	Protein_FDR	Protein_Mass	PeptideSeqs
43_2	tr|D4A4S3|D4A4S3_RAT	7.79248831333	NA	70683.0487347003	FEELNADLFR;IINE
43_1	sp|P06761|BIP_RAT	3.89624415667	NA	72302.4294747003	IINEPTAAAIAYGLI
98_1	sp|B2GUZ5|CAZA1_RAT	13.1621457928	NA	32889.3321847	DVQDSVTVSNEV(

 © 2017 BGI All Rights Reserved.

28_1	tr|A0A0G2JVH1|A0A0G2JVH1_RAT	3.89624415667	NA	212519.5337847	VQVEQQNNGLDV
105_1	sp|P09034|ASSY_RAT	3.89624415667	NA	46466.8984247	GIYETPAGTILYHA
1_1	tr|G3V8C3|G3V8C3_RAT	15.9697154835	NA	53668.0579747001	ETNLESLPLVDTH
103_1	sp|P06762|HMOX1_RAT	2.85485147151	NA	32984.8054447	QRPASLVQDTTS/
89_1	tr|D3ZFQ8|D3ZFQ8_RAT	3.89624415667	NA	35411.9747747	GLLSSLDHTSIR
5_1	tr|Q6GMN8|Q6GMN8_RAT	7.79248831333	NA	102547.3412947	TINEVENQILTR;V(
62_1	tr|Q63747|Q63747_RAT	2.1551026504	NA	24705.0736246999	VLGLVLLR

2 功能分析

2.1 GO注释

Gene Ontology(简称GO)是一个国际标准化的基因功能分类系统，提供了一套动态更新的标准词汇表（Controlled Vocabulary)来全面描述生物体中基因和基因产物的属性。GO总共有三个本体（Ontology），分别描述基因的分子功能（Molecular Function）、细胞组成（Cellular Component）、参与的生物过程（Biological Process）。

详细信息见网站：http://www.geneontology.org.

我们针对鉴定出的所有蛋白进行GO功能注释分析，给出的结果包括两部分：protein2go和go2protein。

protein2go：针对每个蛋白，给出所有相应的GO功能的ID列表。

深圳华大基因股份有限公司 400-706-6615 © 2017 BGI All Rights Reserved.

go2protein：针对三个ontology（cellular component，biological process，molecular function）中所涉及的GO条目，列出所有相应的蛋白的ID及蛋白个数，同时做出统计图，略去没有相应蛋白的GO条目。

搜索 显示

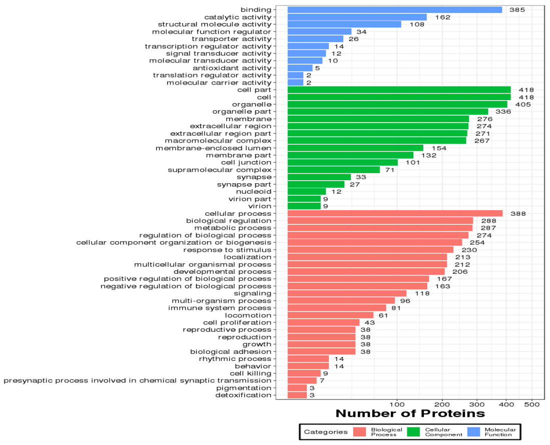

nsun5_co-ip_control

n5_co-iperastin_erastin

图6 GO功能注释图。

GO分类图显示了三个本体中所涉及到各条目的分布情况，不同颜色标记为三个本体中涉及到的各个条目。

nsun5_co-ip_control样本GO注释结果:[nsun5_co-ip_control_GO.zip]

n5_co-iperastin_erastin样本GO注释结果:[n5_co-iperastin_erastin_GO.zip]

@paragraph

2.2 KOG注释

Eukaryotic orthologous groups(KOGs)数据库是一种常用的蛋白功能注释数据库。每个KOG条目都包含一系列直系同源蛋白或旁系同源蛋白(Orthologs or Paralogs)。直系同源蛋白是指来自于不同物种的由垂直家系（物种形成）进化而来的蛋白，并且典型地保留与原始蛋白有相同的功能。旁系同源蛋白是那些在一定物种中的来源于基因复制的蛋白，可能>会进化出新的与原来有关的功能。此次KOG注释分析是将所有鉴定到的蛋白BLAST比对KOG数据库，得到相应的KOG注释结果。

参考文献:\https://www.ncbi.nlm.nih.gov/pubmed/14759257

深圳华大基因股份有限公司 400-706-6615 © 2017 BGI All Rights Reserved.

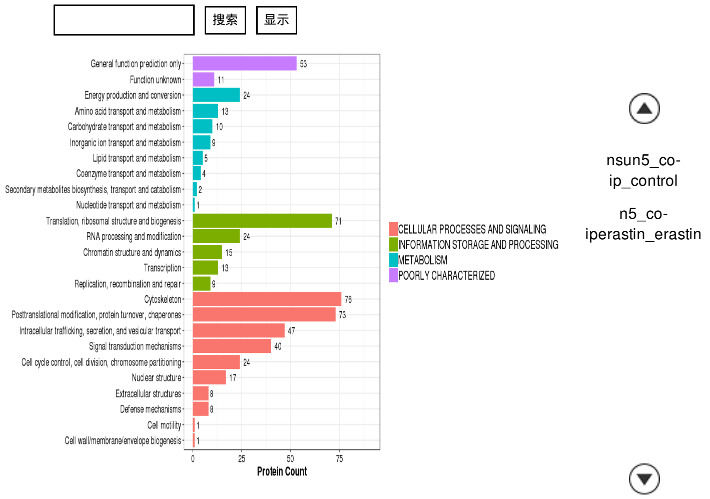

图7 KOG注释柱图。

纵坐标为KOG分类条目，横坐标为功能分类对应的蛋白数量。该图表示样品中不同功能的蛋白质的统计数量。

nsun5_co-ip_control样本KOG注释结果 [nsun5_co-ip_control_KOG.zip]

n5_co-iperastin_erastin样本KOG注释结果 [n5_co-iperastin_erastin_KOG.zip]

2.3 Pathway注释

在生物体内，不同蛋白相互协调行使其生物学行为，基于Pathway的分析有助于更进一步了解其生物学功能。KEGG是有关Pathway的主要公共数据库（Kanehisa，2008），通过Pathway分析能确定蛋白质参与的最主要生化代谢途径和信号转导途径。

以下压缩包为相应样本Pathway注释结果（详见帮助页面中Pathway注释结果说明）:

nsun5_co-ip_control样本Pathway注释结果:[nsun5_co-ip_control_Pathway.zip]

n5_co-iperastin_erastin样本Pathway注释结果:[n5_co-iperastin_erastin_Pathway.zip]

● 分析方法

1 实验流程

蛋白质胶条鉴定，主要是通过凝胶电泳技术对样品蛋白质进行分离，然后分别获取胶片上不同位置的蛋白质胶条，胶内酶解后再抽提出肽段，再运用质谱技术得到这些肽段的质谱图，最后运用蛋白质鉴定软件，鉴定出样品中的蛋白质。

蛋白质胶条鉴定的主要实验步骤如下图所示:

© 2017 BGI All Rights Reserved.

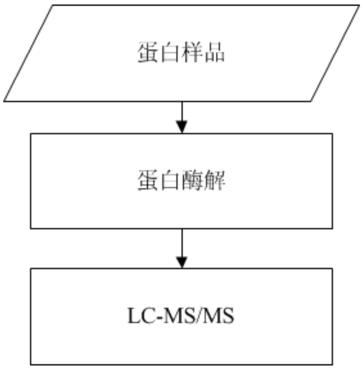

图1 蛋白胶条鉴定的实验流程。

1.1 蛋白酶解

(1) 清洗脱色：考染胶用1ml脱色液（50% 乙腈+ 25mM 碳酸氢铵溶液）清洗多次，直至胶点脱色；银染胶用1ml超纯水清洗3次;

(2) 清洗后的胶点，加入500ul乙腈脱水;

(3) 56 条件下使用10mM DTT（二硫苏糖醇）处理胶点1小时，还原打开二硫键;

(4) 在暗室使用55mM IAM（碘乙酰胺）处理胶点45分钟，进行半胱氨酸的烷基化封闭;

(5) 1ug/ul的酶用25mM碳酸氢铵稀释100倍覆盖胶粒；

(6) 冰上放置30分钟后，补加25mM碳酸氢铵溶液至覆盖胶点，37°C消化过夜;

(7) 转移胶点外面存留的液体到新的离心管;

(8) 用50%乙腈-水从胶点中萃取肽段一次，再用100%乙腈萃取一次;

(9) 两次萃取的溶液和第7步的溶液合并，冷冻抽干。

1.2 高效液相

将抽干的肽段样品用流动相A (2% ACN，0.1%FA)复溶，20,000 g离心10 分钟后，取上清进样。通过岛津公司LC-20AD型号的纳升液相色谱仪进行分离。样品首先进入trap柱富集并除盐，随后与自装C18柱(75微米内径，3.6微米柱料粒径，15厘米柱长)串联，以300 nl/分钟流速通过如下有效梯度进行分离：0-8分钟，5%流动相B(98%ACN，0.1% FA)；8-43分钟，流动相B从8%线性升至35%；43-48分钟，流动相B从35%升至60%；48-50分钟，流动相B从60%升至80%；50-55分钟，80%流动相B；55-65分钟，5%流动相B。纳升液相分离末端直接连接质谱仪。

1.3 质谱检测

经过液相分离的肽段进入到ESI串联质谱仪：TripleTOF 5600(SCIEX, Framingham, MA, USA)，离子源为Nanospray III source(SCIEX, Framingham, MA, USA)，放射器为石英材料拉制的喷针(New Objectives, Woburn, MA，USA)。数据采集时，质谱仪的参数设置如下：离子源喷雾电压2,300V，氮气压力为30psi，喷雾气为15，喷雾接口处温度150摄氏度。采用高灵敏度模式进行扫描，一级质谱扫描累积时间为250ms，扫描质量范围为350-1,500Da。基于一级扫描信息，按照一级谱图中的离子强度从高到低，选择选择强度超过150cp的前30个进行碎裂并扫描二级信息，筛选

© 2017 BGI All Rights Reserved.

标准如下：1)m/z范围为350-1250 Da；2)电荷数目为2-5个电荷；3)母离子动态排除设置为：在一半的出峰时间内(约12s)，相同母离子的碎裂不超过2次。二级质谱的扫描累积时间为100ms。针对iTRAQ类型的数据采集，碎裂能量选择根据iTRAQ试剂调整，第二个四级杆Q2在100Da时的离子传输效率为100%。

2 信息分析流程

蛋白质全谱鉴定主要是通过实验串联质谱数据，与数据库模拟得到的理论质谱数据进行匹配，从而得到蛋白质鉴定结果。流程首先将原始质谱数据转换成质谱峰文件，然后与数据库中的序列进行搜索匹配。并对搜索结果作一些过滤和质量控制，给出可信的蛋白鉴定结果，　　具体过滤参数参考"2.3质量控制"部分。最后基于最终蛋白鉴定列表，完成GO，COG，Pathway等功能注释分析。

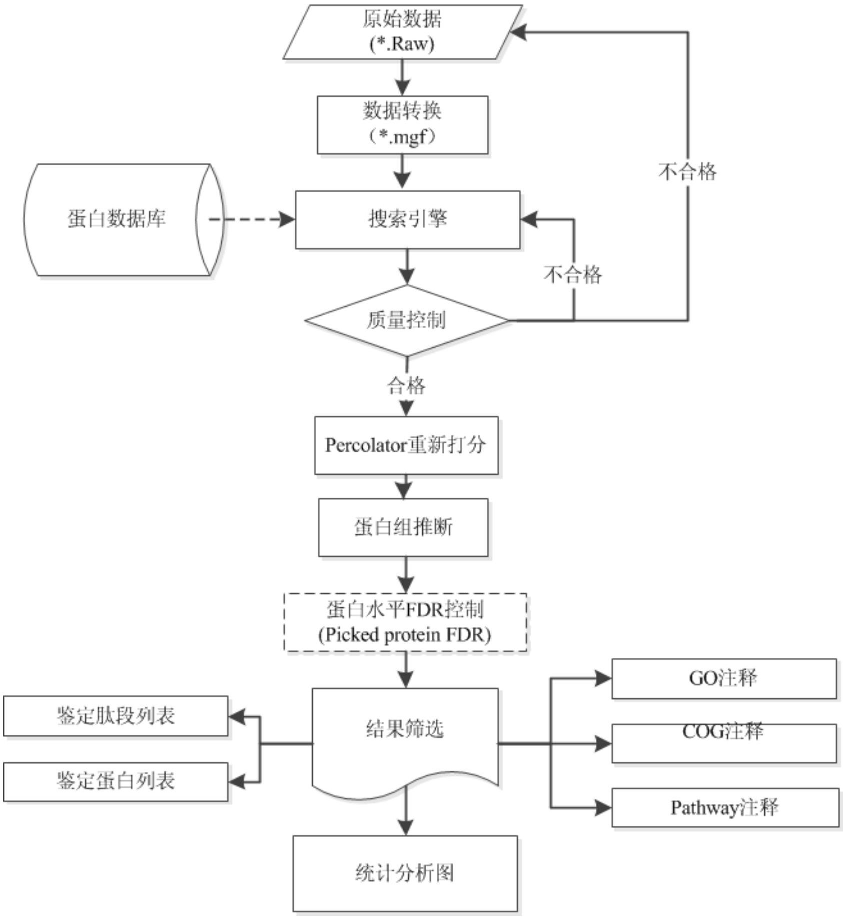

图2 蛋白全谱鉴定的信息分析流程。

2.1 数据库选择

数据库的选择是基于质谱数据的蛋白质鉴定策略中的重要一步，最终鉴定到的蛋白质序列都来源于被选择的数据库。

目前使用到的数据库主要可以分为三大类：

1）UniProt蛋白数据库

UniProt是信息最丰富、资源最广的蛋白质数据库。它由整合Swiss-Prot、 TrEMBL 和 PIR-PSD 三大数据库的数据而成。它是经过专家校验的数据集，主要由两部分组成：UniProtKB/Swiss-Prot

© 2017 BGI All Rights Reserved.

(包含检查过的、手工注释的条目) 和 UniProtKB/TrEMBL (包含未校验的、自动注释的条目)。一般情况下推荐优先使用UniProtKB/Swiss-Prot子集进行相应物种的蛋白质鉴定。当需要寻找新特征序列（如可变剪切，新转录本）或近源物种的鉴定时，才考虑加入UniProtKB/TrEMBL数据库。

2）基于基因组注释的蛋白数据库

主要包括NCBI和Ensembl两大基因注释系统衍生出的一系列数据库。

其中NCBI系统的主要选择参考序列（RefSeq）计划中的蛋白数据库,这是一个非冗余的蛋白质组数据库，由于NCBI注释系统的影响力，该类库广泛应用于多组学关联的分析当中。NCBI的参考序列计划（RefSeq）将为中心法则中自然存在的分子，从染色体到mRNA到蛋白提供参考序列标准。RefSeq标准为人类基因组的功能注解提供一个基础。它们为突变分析，基因表达研究，和多态发现提供一个稳定的参考点。

除此以外，NCBI还提供了非冗余蛋白质序列nr全库(non-redundant)，包括动物、植物、微生物、细菌等分类。由于该数据库来源广泛（包含GeneBank,RefSeq,SwissProt,PDB等等），因此除非是基因组注释不完整的物种，抑或需要进行同源物种的搜索。否则不推荐直接利用该库进行蛋白鉴定。

Ensembl旨在开发一种能够对真核生物基因组进行自动注释(automatic annotation)并加以维护的软件。 Ensembl具有比较完整一致的基因组、转录组、蛋白质组注释信息。非常适合用于涉及多组学关联的分析。

3) 其它来源数据库

通常指客户提供的目标蛋白数据库，或者指基因组转组学测序产生并denovo组装完成的新基因序列。另外还可以包含一些新特征的序列，如可变剪切，突变位点，融合基因等等。

2.2 Mascot搜索

Mascot[1]是蛋白质组学领域重要的蛋白质鉴定软件之一，使用广泛。该项目中使用的软件版本为Mascot2.3.02。操作时以mgf文件为原始文件，选择已经建立好的数据库，然后进行数据库搜索。

本项目鉴定过程中，各参数选择如下：

表1 Mascot搜索参数° （下载）

Item	Value
Search Engine	Mascot v2.3
Enzyme	Trypsin
Peptide Mass Tolerance	0.05Da
Fragment Mass Tolerance	0.1Da
Fixed modifications	Carbamidomethyl (C)
Variable modifications	Oxidation (M); Gln->pyro-Glu (N-term Q); Deamidated (NQ)
Max Missed Cleavages	1
Instrument type	ESI-QUAD-TOF
Database	uniprot_Rattus_norvegicus_nr.fasta (35766 sequences)

2.3 质量控制

本流程将搜索引擎产生的结果使用Percolator\[2\]进行预处理并重新打分，以提高正确匹配和随

© 2017 BGI All Rights Reserved.

机匹配判别的准确性。然后对输出的结果以谱图水平 FDR 1%进行过滤（PSM-level FDR <=0.01），从而获得显著性鉴定的谱图和肽段列表。

接着基于“简约原则”（The parsimony principle），本流程对肽段进行蛋白推断，并产生一系列的蛋白组。

2.4 iBAQ定量

为了获得每个蛋白的iBAQ值。本流程利用华大基因自主开发的软件完成肽段XIC的提取并计算峰面积。然后基于iBAQ算法[3]，将每个蛋白组中总的峰面积除以理论能够观察到的肽段数目，从而获得最终的iBAQ值。

帮助

1 mgf数据格式说明

在生物信息分析中，质谱原始文件需要转换成mgf格式(sample.mgf)后才能被使用。mgf文件主要包含了二级质谱（MS/MS）谱图的信息，其基本格式如下：

```
BEGI NIONS
TITLE=Spectrum1 scans: 2,
PEPMASS=588.84003 11629.05371
CHARGE=2+
RTINSECONDS=0
SCANS=2
115.55481 258.814
116.11040 496.234
136.94980 420.235
173.90498 377.256
180.86188 368.726
190.94720 352.431
………
END IONS
```

其中“BEGIN IONS”和“END IONS”是每一张谱的开始和结束位置，“TITLE”为该谱图的编号信息，“PEPMASS”为母离子质荷比和强度值，“CHARGE”为母离子所带电荷数，“RTINSECONDS”为保留时间，“SCANS”为扫描编号，其余各行为肽段经碎裂后的子离子质荷比值和强度信息。

更多关于mgf格式的说明，请访问网址 http://www.matrixscience.com/help/data_file_help.html。

2 数据目录编排说明

由于报告展示篇幅有限，完整的数据可通过网页报告侧边栏“文件--Download the Files”下载至本地。各主要目录结果的编排及说明如下图：

深圳华大基因股份有限公司 400-706-6615 © 2017 BGI All Rights Reserved.

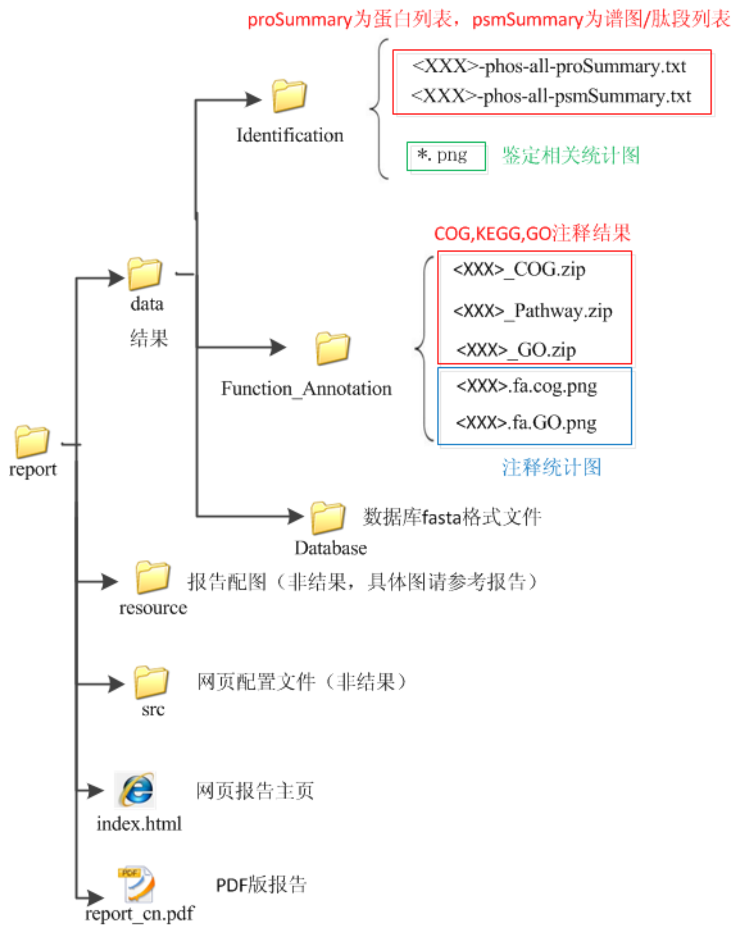

图1 结果目录结构。

3 蛋白质序列FASTA格式

基于文本的FASTA格式文件用于存储记录DNA或是蛋白质序列信息。序列文件的第一行以符号“>”开头，紧接着序列ID，之后可以跟随序列注释信息。第二行为第一行ID对应的DNA碱基序列或者蛋白质氨基酸序列。FASTA文件可用写字板直接打开查看。

举例如下：

```
>tr|F2N3W2|F2N3W2_PSEU6 Quinoprotein alcohol dehydrogenase OS=Pseudomonas stutzeri (strain DSM 4166 / CMT.9.A) GN=PSTAA_2293 PE=4 SV=1
MKHTGLRKPFAVTALCAAVAMSSLHAWAVTDQEILNDAKSTDQIVTNGLGLQGQRYSTLD
ALNTNNINQLRPVWGFSLGGEKQRGQEAQPLIKDGVMYITGSYSRVYALDARTGKELWQ
YDARLPDGIMPCCDVINRGVALYDDLVIFGTLDAKLVALNKDTGKVVWKKTVADYKAGY
SLTAAPLVVN
```

4 peptideSummary文件格式说明

该文件包含本次项目所有样本鉴定到的谱图和肽段相关信息。每列以tab符号分隔，可使用Excel打开。

表1 谱图和肽段信息格式说明 （下载）

深圳华大基因股份有限公司 400-706-6615 © 2017 BGI All Rights Reserved.

Field	Description
query_number	谱图号
peptide_seq	肽段序列
PSM_FDR	谱图水平FDR, 即通常文章中所指的"蛋白FDR"（非蛋白水平FDR）
ions_score	谱图匹配得分
Protein_ID	相应蛋白ID

5 proteinSummary文件格式说明

该文件记录了样品鉴定到的蛋白质的信息列表。每列以tab符号分隔，可使用Excel打开。

表2 蛋白信息格式说明 （下载）

Field	Description
Group_ID	蛋白组序号
Protein_ID	蛋白组首ID
Protein_Qscore	蛋白得分
Protein_FDR	根据Protein_Qscore计算获得的蛋白水平FDR(只有启用Protein-level FDR过滤模式本列才有值，否则为NA)
Protein_Mass	蛋白质量
PeptideSeqs	蛋白中鉴定到的肽段序列（以;分开）
PepIsUnique	与PeptideSeqs列中一一对应的肽段是否为Unique肽段，1为Unique,0为非Unique.
Unique_Peptide_Num	该蛋白组中Unique肽段数目。
Unique_Spectra_Num	该蛋白组中Unique谱图数目。
Coverage	蛋白覆盖度
Peptide_Query	支持该蛋白组的谱图号
Sameset	蛋白组中其余蛋白ID
Description	蛋白描述
Abundance	蛋白丰度值（需要启用iBAQ定量才有这栏）
iBAQ	蛋白iBAQ定量值（需要启用iBAQ定量才有这栏）

6 GO功能注释说明

GO功能注释结果打包于各样品目录下的sample_GO.zip压缩包中，sample.fa.GO.png 为GO 分类图文件，sample.fa.GO2protein.xls 为 GO 分类文件（GO 条目与蛋白质的对应关系），该文件可以使用 Excel打开。

	A	B	C	D	E	F
1	Ontology	Class	number_of_Nb	proteins_of_Nb;		
2	biological_process	biological regulation	101	Niben101Scf03422g01002.1;		

图2 GO2protein示例图。

表3 GO2protein格式说明 （下载）

Field	Description
Ontology	GO本体的类别(biological_process 或 cellular_component 或 molecular_function)
Class	GO条目
number_of_*	注释到各GO条目中的蛋白质数量
Proteins_of_*	注释到各GO条目蛋白质ID

© 2017 BGI All Rights Reserved.

而sample.fa.protein2GO.xls 中为 GO 注释（蛋白质与 GO 条目的对应关系）文件，该文件可以使用 Excel打开。

	A	B	C	D
1	Niben101Ctg05133g00002.1	GO:0008614	GO:0009108	

图3 protein2GO示例图。

表4 protein2GO格式说明 （下载)

Field	Description
第一列	蛋白质ID
第二列及以后	GO ID

7 COG功能注释说明

COG注释结果打包于各样品目录下的sample_COG.zip压缩包中，sample.cog2protein.xls为COG分类文件（COG 条目与蛋白质的对应关系），该文件可以使用 Excel打开。

	A	B	C	D	E
1	Code	Functional-Categories	Protein-Number	Proteins	
2	A	RNA processing and modi:	1	Niben101Scf07457g0	
3	B	Chromatin structure and	3	Niben101SNiben101S	
4	C	Energy production and c	124	Niben101SNiben101S	

图4 COG2protein示例图。

表5 COG2protein格式说明 （下载)

Field	Description
Code	COG功能代号
Functional-Categories	COG功能分类
Protein-Number	蛋白质在各COG功能分类的数量
Proteins	蛋白质在各COG功能分类的序列ID

而sample.protein2COG.xls 中为COG注释（蛋白质与COG条目的对应关系）文件，该文件可以使用 Excel打开。

	A	B	C	D	E	F	G	H	I
1	Protein	Protein-or-Domain	Score	E-Value	COG-ID	Function-Descript	Code	Functional-Categories	
2	Niben101Scf1	CT539	95.1	3.00E-20	COG0526	Thiol-disulfide i	OC	Posttranslational moc	
3	Niben101Scf1	CPn0659	93.2	1.00E-19	COG0526	Thiol-disulfide i	OC	Posttranslational moc	

图5 protein2COG示例图。

表6 protein2COG格式说明 （下载)

Field	Description
Protein	蛋白质序列的名称
Protein-or-Domain	比对上的蛋白或结构域
Score	blast比对的得分
E-Value	blast比对的E-value
COG-ID	COG的ID
Function-Description	功能描述
Code	COG功能代号
Functional-Categories	COG功能分类

深圳华大基因股份有限公司 400-706-6615 © 2017 BGI All Rights Reserved.

8 Pathway通路注释说明

Pathway结果打包于各样品目录下的sample_Pathway.zip压缩包中，sample.ko为蛋白ID及对应KO号列表文件（该文件可以选择 Excel用打开，也可以用终端less命令查看）。

	A	B	C	D	E	F
1	# Method: BLAST	Condition: expect <= 1e-100; rank <= 5				
2	# Summary:	147 succeed, 722 fail				
3						
4	# query	ko_id:rank:evalue:score:identity:ko_definition				
5	Niben101Ctg05133g00002.1					
6	Niben101Ctg08688g00001.1					
7	Niben101Ctg12954g00001.1					
8	Niben101Ctg13092g00001.1					
9	Niben101Ctg14629g00001.1					
10	Niben101Ctg15034g00003.1					
11	Niben101Ctg16053g00004.1	K13811|1|3e-117|424|mze:101464984|EC[not_get]				
12	Niben101Ctg16210g00007.1	K08912|1|6e-110|399|rro:104672455|EC[not_get]				
13	Niben101Ctg16210g00008.1					
14	Niben101Scf00022g06009.1					

图6 *.ko示例图。

表7 *.ko格式说明 （下载)

Field	Description
第一至三行 分析过程信息说明	
第一列 蛋白	
第二列 KEGG Orthology	

sample.path 为蛋白的 Pathway 列表文件（该文件可以选择 Excel用打开，也可以用终端less命令查看）。

	A	B	C	D	E
1	#Pathway	Count (14	Pathway ID	Proteins	KOs
2	Metabolic pathways	83	ko01100	Niben101C	K13811+K08
3	Biosynthesis of seco	52	ko01110	Niben101S	K00600+K01
4	Microbial metabolism	47	ko01120	Niben101C	K13811+K0(
5	Biosynthesis of anti	47	ko01130	Niben101C	K13811+K0(
6	Carbon metabolism	38	ko01200	Niben101S	K00600+K0(
7	Biosynthesis of amin	25	ko01230	Niben101S	K00600+K01
8	Glyoxylate and dicar	18	ko00630	Niben101S	K00600+K0(
9	Glycolysis / Glucone	17	ko00010	Niben101S	K00128+K0(
10	Pyruvate metabolism	13	ko00620	Niben101S	K00026+K0(

图7 *.path示例图。

表8 *.path格式说明 （下载)

Field	Description
Pathway	通路名
Count	注释到该通路的蛋白质的数目
Pathway ID	KEGG数据库中的Pathway ID
Proteins	注释到该Pathway的蛋白
KOs	属于该Pathway的KEGG Orthology

sample_map文件夹中包含蛋白的 Pathway图文件。sample.htm为蛋白的Pathway分析结果文件,用浏览器打开查看。

© 2017 BGI All Rights Reserved.

1. Nb

#	Pathway	Diff Proteins with pathway annotation (147)	Pathway ID
1	Metabolic pathways	83 (56.46%)	ko01100
2	Biosynthesis of secondary metabolites	52 (35.37%)	ko01110
3	Microbial metabolism in diverse environments	47 (31.97%)	ko01120
4	Biosynthesis of antibiotics	47 (31.97%)	ko01130
5	Carbon metabolism	38 (25.85%)	ko01200
6	Biosynthesis of amino acids	25 (17.01%)	ko01230
7	Glyoxylate and dicarboxylate metabolism	18 (12.24%)	ko00630
8	Glycolysis / Gluconeogenesis	17 (11.56%)	ko00010
9	Pyruvate metabolism	13 (8.84%)	ko00620
10	Citrate cycle (TCA cycle)	13 (8.84%)	ko00020

图8 *.htm示例图。

表9 *.htm格式说明 （下载）

Field	Description
Pathway	通路名，点击可看到注释到该通路的各蛋白质情况及Pathway图
Diff Proteins with pathway annotation	鉴定的蛋白质注释到该通路的数目及比例
Pathway ID	KEGG数据库中的Pathway ID

● 常见问题

蛋白鉴定列表中Protein_FDR那列的值是NA意味着什么？

通常对于蛋白鉴定数目较多的项目，为了保证结果的严谨性，我们利用Picked protein FDR算法重新计算蛋白FDR，从蛋白水平更严格的进行鉴定结果的筛选，此时Protein_FDR列会有相应的FDR值。而对蛋白鉴定数目较少的项目（比如胶条鉴定），通常我们只进行谱图水平的FDR控制，此时过滤阈值会比较宽松，保证鉴定数目不致过少，因此，此时的Protein_FDR列不会有相应的值，相应只会显示NA。

● 参考文献

[1] Perkins, D. N.; Pappin, D. J.; Creasy, D. M.; Cottrell, J. S., Probabilitybased protein identification by searching sequence databases using mass spectrometry data. Electrophoresis 1999, 20, 35513567.

[2] Lukas Käll, Jesse Canterbury, Jason Weston, William Stafford Noble and Michael J. MacCoss. "Semi-supervised learning for peptide identification from shotgun proteomics datasets." Nature Methods. 4(11):923-925, 2007.

[3] Schwanhäusser B, Busse D, Li N, Dittmar G, Schuchhardt J, Wolf J, Chen W, Selbach M: Global quantification of mammalian gene expression control. Nature 2011, 473: 337–342.

2015 Copyright BGI All Rights Reserved 粤ICP备 12059600
Technical Support E-mail:info@bgitechsolutions.com
Website: www.bgitechsolutions.com

© 2017 BGI All Rights Reserved.

基因科技造福人类

联系我们

服务热线：400-706-6615
网址：www.bgitechsolutions.com
邮　箱：info@bgitechsolutions.com
地址：广东省深圳市盐田区北山工业区11栋（邮编：518083）

本结题报告仅供客户学习、交流和研究使用，请勿用于商业用途，违者必究。
版权声明：本结题报告版权属于深圳华大基因股份有限公司所有，未经本公司书面许可，任何其他个人或组织均不得以任何形式将本结题报告中的各项内容进行复制、拷贝、编辑或翻译为其他语言。本结题报告中的所有商标或标志均属于深圳华大基因股份有限公司及其提供者所有。
2017年01月
