# Supplementary figures and images for: The NSUN5-FTH1/FTL pathway mediates ferroptosis in bone marrow-derived mesenchymal stem cells
Source: Cell Death Discov. 2022 Mar 5;8:99. doi: 10.1038/s41420-022-00902-z (PMC8898311; doi:10.1038/s41420-022-00902-z)

A

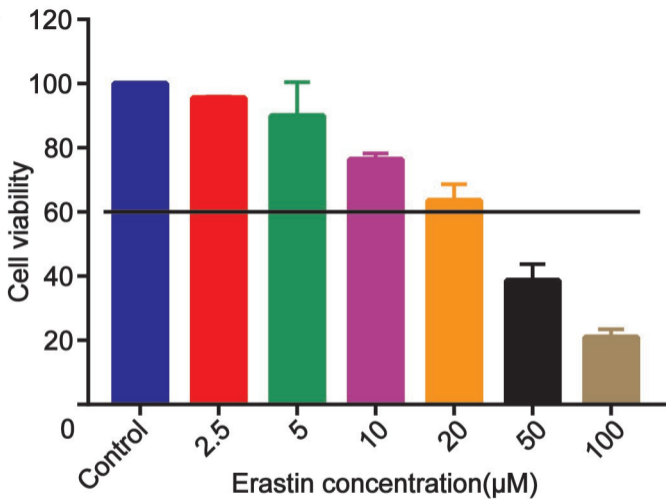

Supplement: Supplementary file 4 — Supplementary Figure 1 [file 41420_2022_902_MOESM4_ESM.pdf]

**A**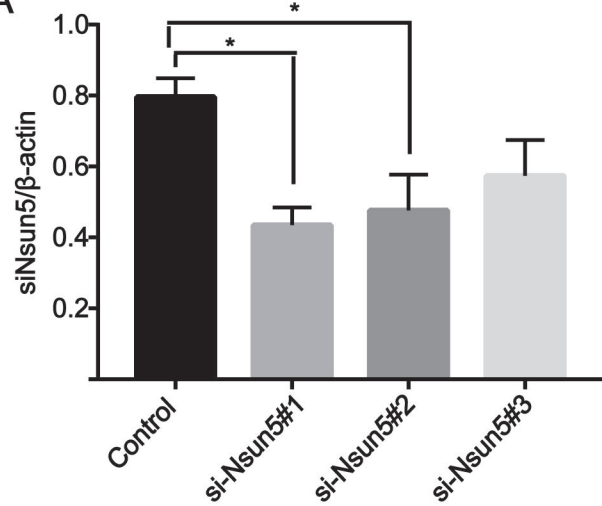**B**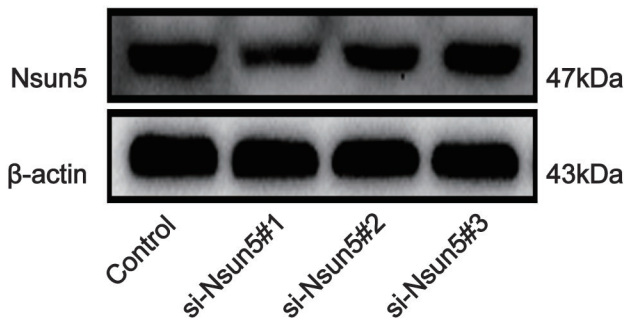**C**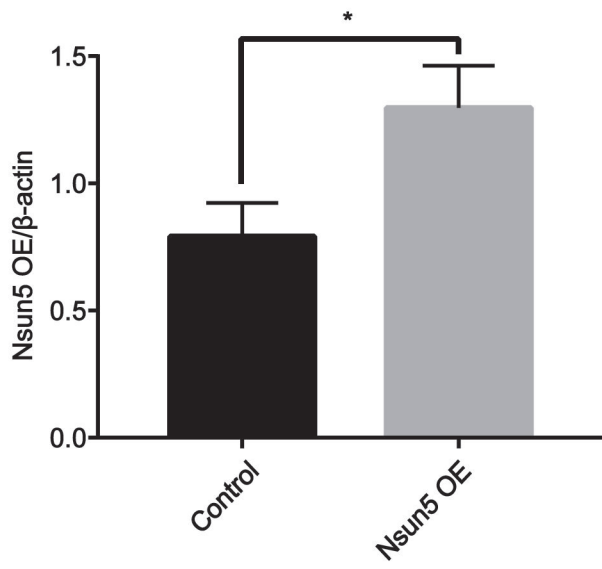**D**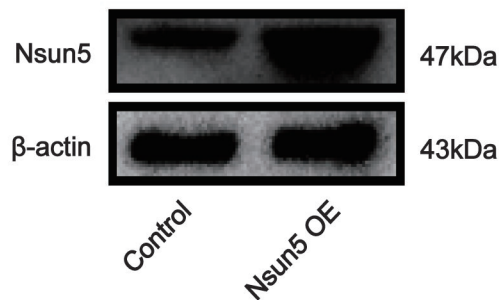

Supplement: Supplementary file 5 — Supplementary Figure 2 [file 41420_2022_902_MOESM5_ESM.pdf]

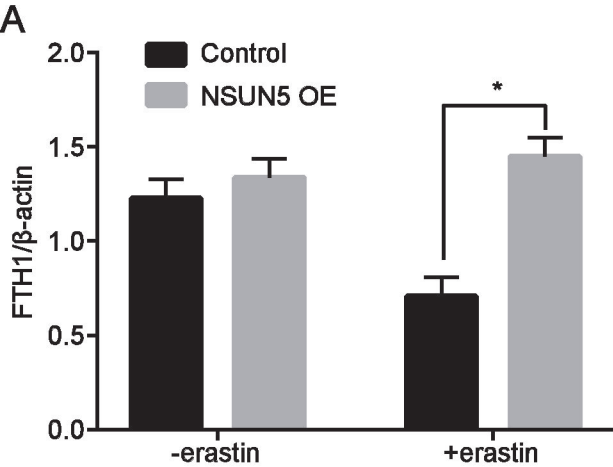

The quantify of figure 5 C

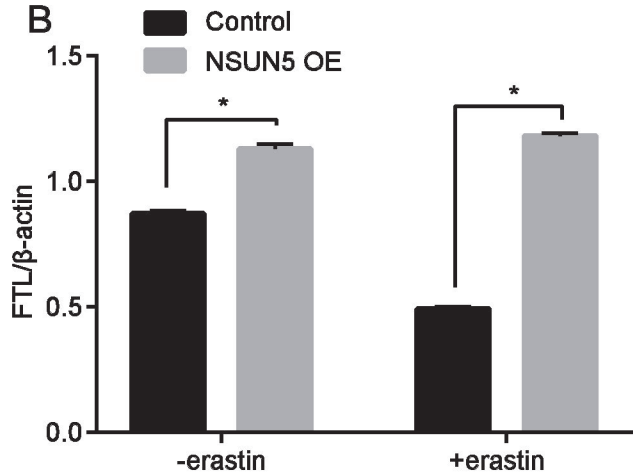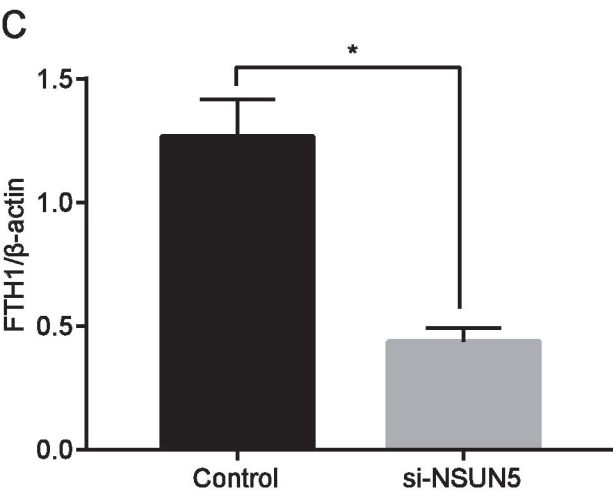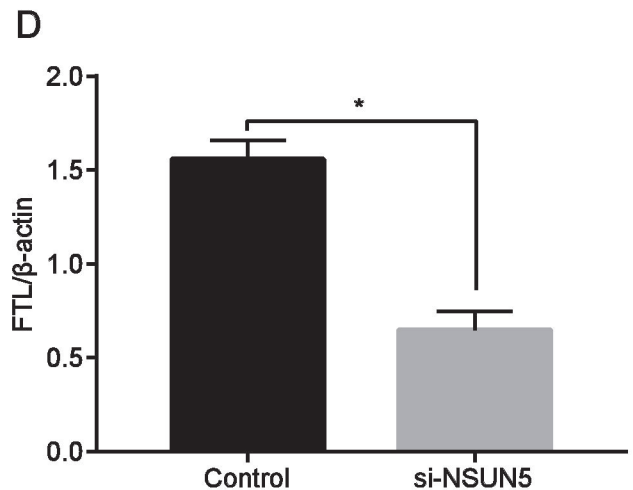

The quantify of figure 5 D

Supplement: Supplementary file 6 — Supplementary Figure 3 [file 41420_2022_902_MOESM6_ESM.pdf]

**E**

**Control**

**DAPI**

**Nsun5**

**FTL**

**FTH1**

**Merge**

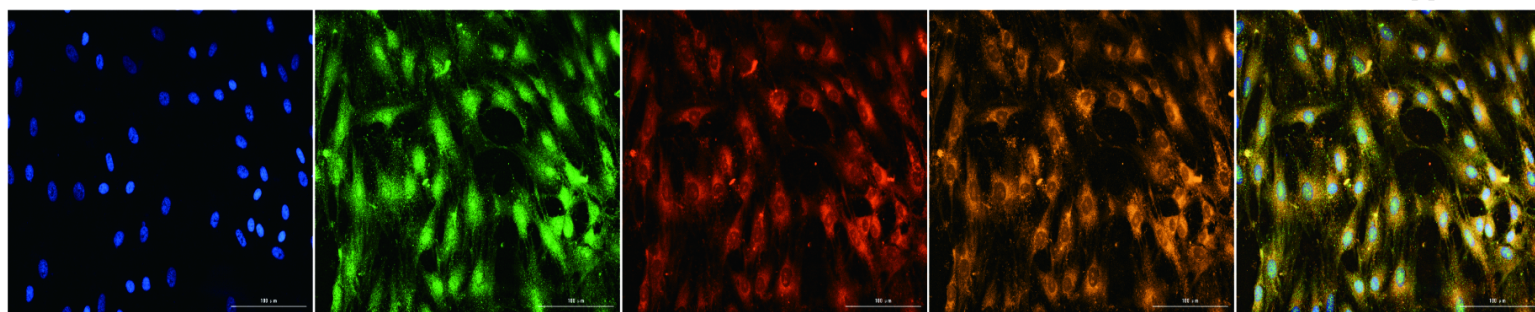

**Erastin**

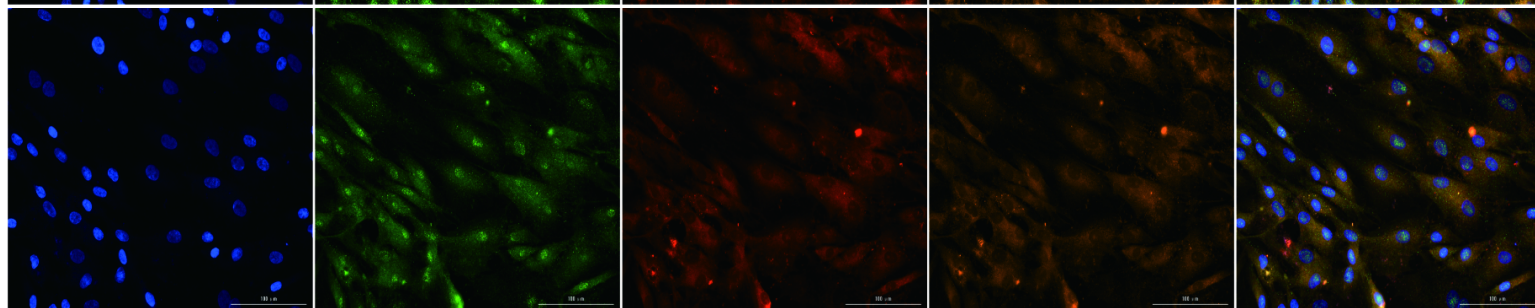

Supplement: Supplementary file 7 — Supplementary Figure 4 [file 41420_2022_902_MOESM7_ESM.pdf]

Control

Erastin

DAPI

Nsun5

FTL

FTH1

Merge

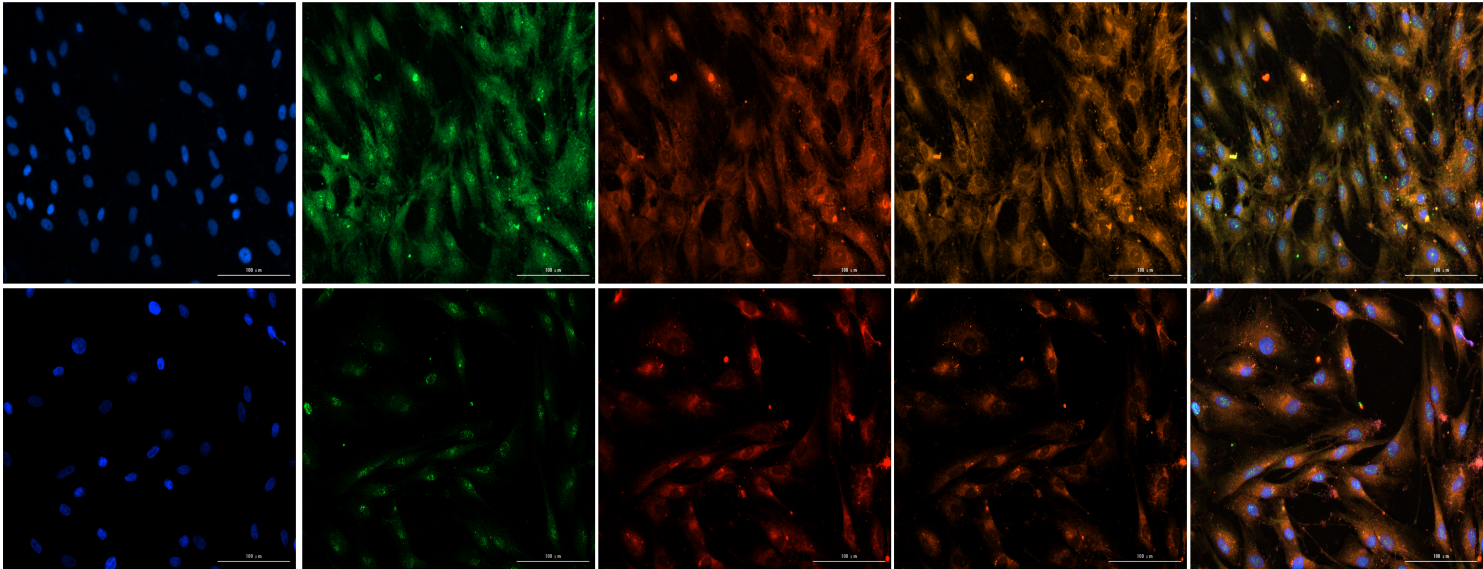

Supplement: Supplementary file 8 — Supplementary Figure 5 [file 41420_2022_902_MOESM8_ESM.pdf]

**A**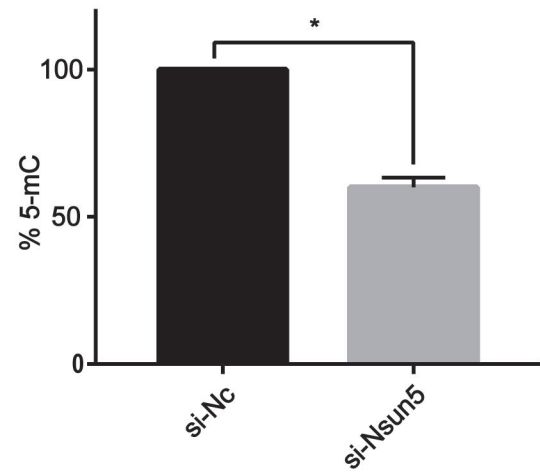**B**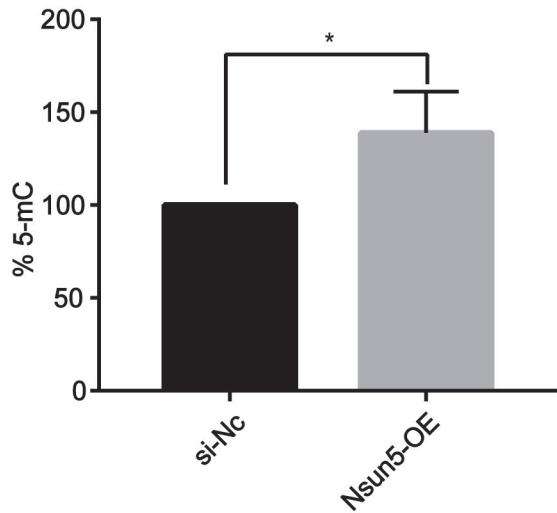

The quantify of fig 5 A, B

Supplement: Supplementary file 9 — Supplementary Figure 6 [file 41420_2022_902_MOESM9_ESM.pdf]

A

Input IgG IP:NSUN5

IB: Trap1

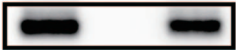

Input IgG IP:Trap1

IB: NSUN5

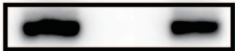

Supplement: Supplementary file 10 — Supplementary Figure 7 [file 41420_2022_902_MOESM10_ESM.pdf]

C

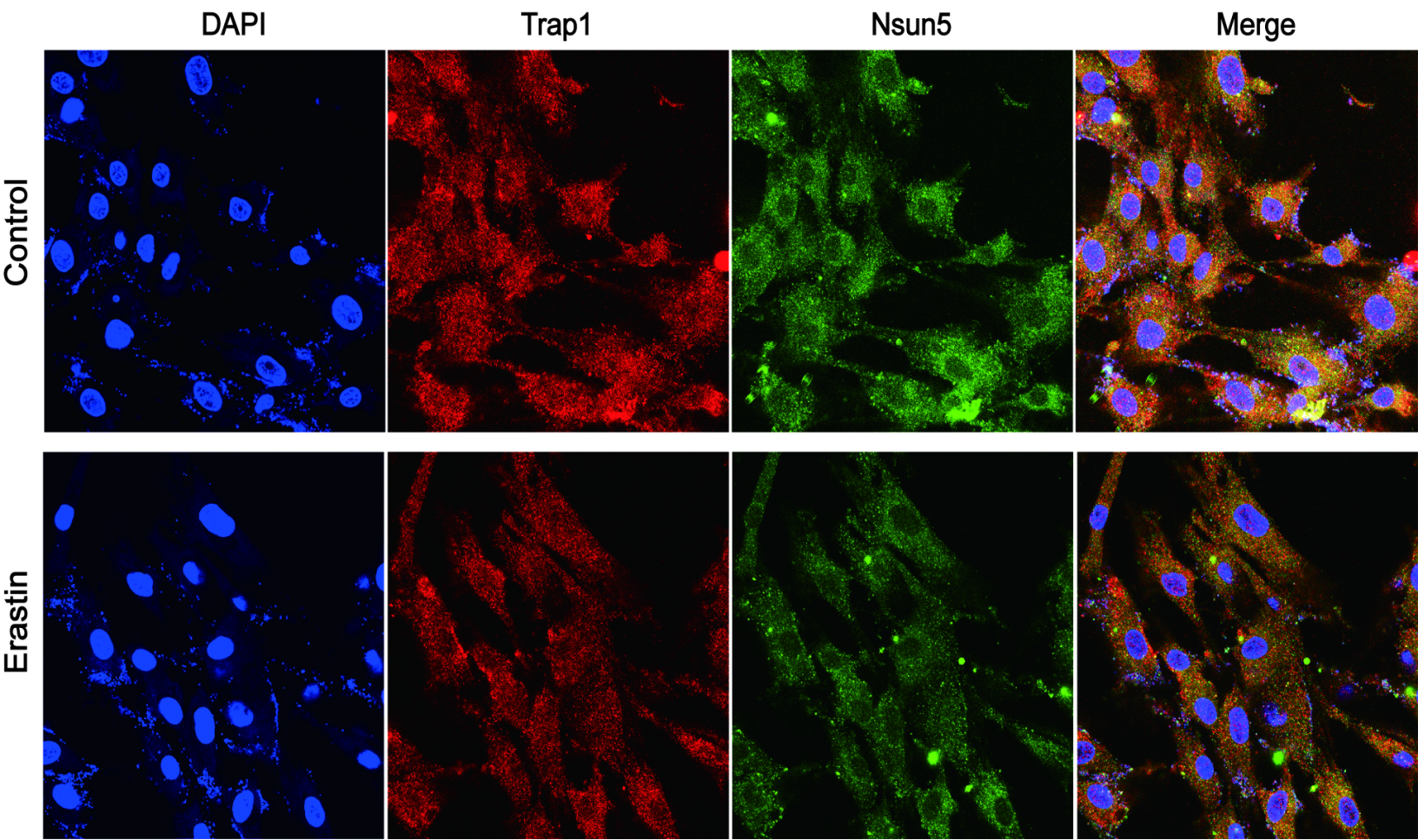

Supplement: Supplementary file 11 — Supplementary Figure 8 [file 41420_2022_902_MOESM11_ESM.pdf]

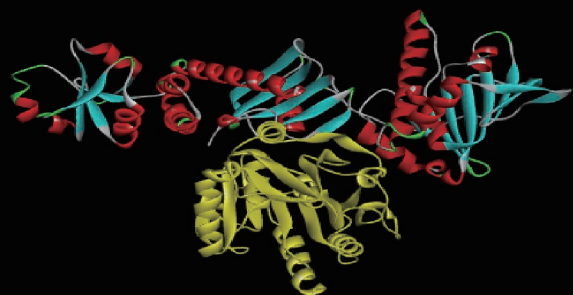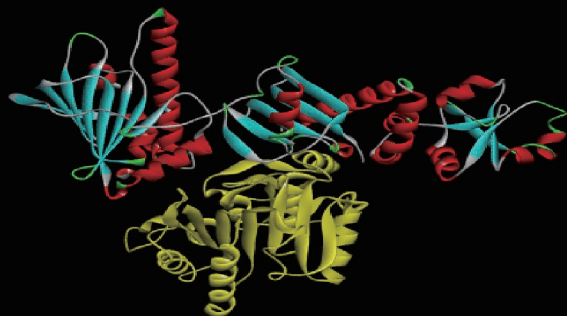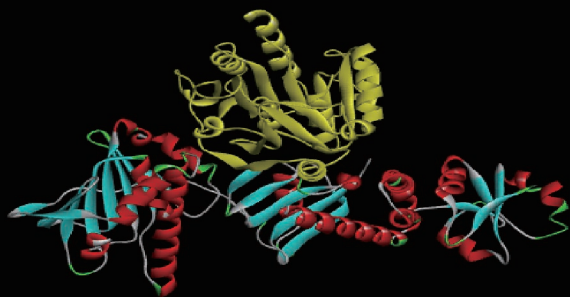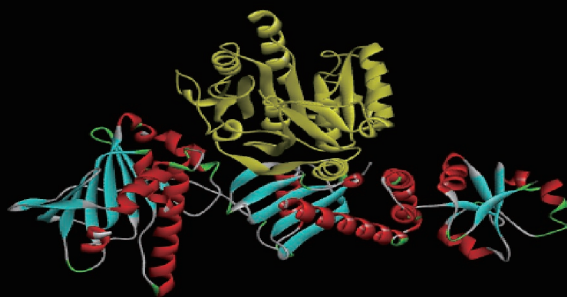

Supplement: Supplementary file 12 — Supplementary Figure 9 [file 41420_2022_902_MOESM12_ESM.pdf]
